# Interleukin-1 Ligands and Receptors in Lumpfish (*Cyclopterus lumpus* L.): Molecular Characterization, Phylogeny, Gene Expression, and Transcriptome Analyses

**DOI:** 10.3389/fimmu.2020.00502

**Published:** 2020-04-02

**Authors:** Håvard Ø. Eggestøl, Harald S. Lunde, Tim Martin Knutsen, Gyri T. Haugland

**Affiliations:** ^1^Department of Biological Sciences, Bergen High-Technology Centre, University of Bergen, Bergen, Norway; ^2^Aquagen AS, Trondheim, Norway

**Keywords:** IL-1 beta, IL-18, nIL-1F, IL-1R, lumpsucker, innate immune responses, inflammation

## Abstract

The interleukin (IL)-1 family play a fundamental role as immune system modulators. Our previous transcriptome-analyses of leukocytes from lumpfish (*Cyclopterus lumpus* L.) showed that IL-1β was among the most highly upregulated genes following bacterial exposure. In the present study, we characterized IL-1 signaling pathways, identified and characterized four ligands of the IL-1 family in lumpfish; IL-1β type I and type II, IL-18, and the novel IL-1 family members (nIL-1F), both at mRNA and gene levels. The two IL-1β in lumpfish is termed IL-1β1 (type II) and IL-1β2 (type I). Furthermore, a comprehensive phylogenetic analysis of 277 IL-1 ligands showed that nIL-1F, in common with IL-1β, likely represents an ancestral gene, as representatives for nIL-1F were found in cartilaginous and lobe-finned fish, in addition to teleosts. This shows that nIL-1F is not exclusively present in teleosts as previously suggested. Our analyses of exon-intron structures, intron phases, phylogeny and synteny clearly show the separation of IL-1β into groups; type I and type II, which likely is a result of the third whole genome duplication (3R WGD). The phylogenetic analysis shows that most teleosts have both type I and type II. Furthermore, we have determined transcription levels of the IL-1 ligands in leukocytes and 16 different tissues, and their responses upon *in vitro* stimulation with seven different ligands. In addition, we have identified the IL-1 receptors IL-1R1, IL-1R2, IL-1R4 (ST2/IL-33 receptor/IL-1RL), IL-1R5 (IL-18R1), and partial sequences of DIGIRR and IL-1R3 (IL-RAcP). Identification of immune molecules and description of innate responses in lumpfish is interesting for comparative and evolutionary studies and our study constitutes a solid basis for further functional analyses of IL-1 ligands and receptors in lumpfish. Furthermore, since lumpfish are now farmed in large numbers to be used as cleaner fish for removal of sea lice on farmed salmon, in-depth knowledge of key immune molecules, signaling pathways and innate immune responses is needed, as the basis for design of efficient immune prophylactic measures such as vaccination.

## Introduction

Cytokines belonging to the IL-1 family are key mediators of the body's response to microbial invasion, inflammation, immunological reactions, and tissue injury. In mammals, the IL-1 family consists of 11 cytokines. Of these, seven have pro-inflammatory activity (IL-1α, IL-1β, IL-18, IL-33, IL-36α, IL-36β, and IL-36γ), three are antagonistic (IL-1Ra which is also known as IL-1RN, IL-36RN, and IL-38) and one has anti-inflammatory properties (IL-37) (1). Of these, only IL-1β and IL-18, also referred to as IL-1F2 and IL-1F4, respectively, have been identified in teleosts thus far. Fish have, however, multiple paralogs of many cytokines (2) and multiple IL-1β have been identified in several fish species, including channel catfish (3), salmon, trout (4, 5) and carp (3, 6). The genes encoding IL-1β in teleost fish are divided into two groups (type I and II) based on the number of exon/intron and synteny analyses (4, 7). Type I has been identified in species belonging to Neoteleostei and Protacanthopterygii, e.g., carp, cod, salmon, and in the most evolutionary advanced fishes, such as gilthead seabream (*Sparus aurata*), European seabass, three-spined stickleback, Nile tilapia, southern platyfish, Japanese rice fish and Japanese flounder (8). IL-1β2 show low sequence identity with IL-1β type I, but contain the IL-1 family signature [FC]-x-S- [ASLV]-x(2)- [FYLIV]- [LI]- [SCA]-T-x(7)- [LIVM] (prosite, PDOC00226) and a β-trefoil structure with β-sheets. Type II has thus far been identified in many fish species, but not in species belonging to Cypriniformes (4, 7). Gene expression studies of IL-1β have shown that it is significantly upregulated in immune tissues, in primary cultures and cell lines in response to immunostimulants, immune response modifiers and/or pathogens [reviewed in (2)].

IL-18 contains an IL-1 like signature sequence and has, like IL-1β, 12 β-sheet strands that form a β-trefoil structure (9). IL-18 has been described in trout and identified in other species like Japanese—and green spotted pufferfish (10) and seabass (2). In trout, like in mammals, IL-18 is constitutively expressed in a wide range of tissues (10). Transcription of IL-18 is not modulated by LPS, poly(I:C) or trout recombinant IL-1β in head kidney leukocytes (HKL) and a macrophage cell line. A shorter, alternative spliced transcript of IL-18 is, on the other hand, upregulated in RTG-2 cells (a fibroblast cell line from rainbow trout) after stimulation with LPS or poly(I:C), suggesting that proteolytic cleavage may be crucial for mediating a biologically active IL-18 in fish, as in mammals (7). Mammalian IL-18 has multiple functions in both innate and adaptive immunity, such as induction of IFN-γ in Th1 and NK cells, promotion of T and NK cell maturation and neutrophil activation. Recently, it was also shown that IL-18 is also involved in Th2 responses (11). In fish, the functions of IL-18 are less understood (10).

The teleost specific IL-1 family member is termed novel IL-1 family members (nIL-1F, also known as nIL-1Fm) (8, 12–14). nIL-1F has been identified in three-spined stickleback (*Gasterosteus aculeatus*), European seabass (*Dicentrarchus labrax*), Japanese flounder (*Paralichthys olivaceus*), Nile tilapia (*Oreochromis niloticus*), southern platyfish (*Xiphophorus maculatus*), Japanese puffer (*Takifugu rubripes*), spotted green pufferfish (*Tetraodon nigroviridis*), Japanese rice fish (*Oryzias latipes*), rainbow trout (*Oncorhynchus mykiss*), zebrafish (Danio rerio), channel catfish (*Ictalurus punctatus*), and grass carp (*Ctenopharyngodon idella*) (8, 12–14). nIL-1F also have the IL-1 family signature and the β-trefoil structure with β –sheets. It has been suggested that nIL-1F antagonizes IL-1β activity similar to IL-1Ra in mammals (13–15).

In mammals, IL-1β and IL-18 are produced as pro-peptides and require proteolytic cleavage for activation. The IL-1β precursor is cleaved typically with cytosol caspase-1/interleukin-1 converting enzyme (ICE) between the aspartate amino acid at position 116 and alanine at position 117 to form mature proteins. Alternatively, pro-IL1β can also be cleaved by enzymes like granzyme A, trypsin, chymase, elastase, cathepsin G, collagenase, matrix metalloproteases, or serine proteases (2). Fish and other non-mammalian species do not have the conserved Asp^116^. Caspases are, however, also involved in processing of IL-1 β in fish. In zebrafish, Caspases A and B cleave IL-1β at position D104 and D122. Alternatively, zebrafish caspase B cleaves IL1β at position D88 instead of D104 (16). Sea bass caspase-1 cleaves proIL-1β at D100 (17). Gilthead seabream IL-1β is processed before being released, but the mechanism is not known as it does not have a conserved caspase-1 processing site and neither inhibitors of pan-caspase nor caspase-1 inhibit its processing (8). It has been suggested that IL-18 in fish, as in mammals, needs proteolytic processing to become a functionally active protein. Potential ICE cut sites have been predicted for IL-18 and nIL-1F, but functional analyses are required to verify this (9, 13, reviewed in 7).

The IL-1 receptor (IL-1R) family comprises 10 members and includes cytokine-specific receptors, co-receptors and inhibitory receptors (18–20). A novel receptor nomenclature has been proposed by Boraschi et al. (18) and will be followed here. There are two types of receptors that bind mammalian IL-1α/β: type I IL-1 receptor (IL-1R1) which binds to the accessory receptor protein IL-1R3 (also known as IL-1RAcP and IL-1RAP) and type II IL-1 receptor (IL-1R2), which also binds to IL-1R3. IL-1R2 is a decoy receptor that is structurally incapable of signaling (21) due to the lack of an intracellular domain. Thus, IL-1R2 serves as a negative regulator, both by competing with IL-1R1 for IL-1β and by complexing with IL-1R3, preventing dimerization of IL-1R3 with IL-1R1. Interaction between IL-1R1 and IL-1R3 is needed for downstream signaling from IL-1R1 after IL-1β binding. Both IL-1R1 and IL-1R2, in addition to IL-1R3 are described in fish, such as miiuy croaker (*Miichthys miiuy*), grass carp, Atlantic salmon (*Salmo salar*), orange-spotted grouper (*Epinephelus coioides*), gilthead seabream, rainbow trout, Japanese flounder [(22–24), reviewed in 7]. The IL-18 receptor in mammals consists of two subunits, IL-1R5 (also known as IL18Rα) and IL-1R7 (IL-18Rβ), while in fish, only one IL-18 receptor, IL-18R1, has been described thus far (7). In mammals, the soluble IL-18-binding protein (IL-18BP) regulates IL-18 activity (25). In grass carp, IL1R8, two isoforms of IL1R9 and IL1R10 have also been described (24). nIL-1F binds to the type I IL-1β receptor competing with IL-1β (14).

The aim of the current study was to identify and characterize IL-1 family ligands and receptors in lumpfish, as well as the signaling pathways NF-κB and MAPK to gain further insight into the role of the IL-1 family in innate immunity. In addition, a comprehensive phylogenetic analysis was performed to investigate the evolution of IL-1 ligands.

## Materials and Methods

### Fish and Rearing Conditions

Unvaccinated, farmed lumpfish were provided by Fjord Forsk Sogn AS, a commercial breeder in Sogn & Fjordane County, Norway and kept in a 500 L tank in the rearing facilities at the Industrial and Aquatic Laboratory (ILAB) at Bergen High-Technology Centre under normal optimal rearing conditions, with an average temperature of 10.7 ± 1.7°C, oxygen level of 88 ± 6.4 % (*n* = 147 days), salinity of 34 PPT and light regime 12 h light: 12 h dark. The fish were fed with dry commercial feed [Gemma Silk (3 mm) Skretting, Norway].

### Tissue Sampling and Homogenization

The fish (282.7 ± 56.5 g and 18.5 ± 1.1 cm) were randomly selected and killed by a sharp blow to the head, which is an appropriate procedure under Norwegian law. Peripheral blood (0.7 mL) was collected from the *vena caudalis* and transferred to heparinized containers. Skin mucus was harvested by scraping a sterile scalpel blade along the most lateral skin on the left side of the fish. The skin sample was dissected from a square, were the cranial-ventral corner touched the third skin knot. The muscle sample was harvested from the underlying white musculature. Thymus was harvested by scraping the most cranial-dorsolateral surface tissue of the mouth cavity with a sterile scalpel. Gill tissues, sampled from the second gill arch on the left side, consisted of filaments from the most caudal point on the gill arch. The gill arch sample consisted of the most proximal millimeter of the gill filament, intra-branchial tissue and the gill arch. The tongue, liver, spleen, pyloric caeca, heart and gut were dissected out aseptically. The posterior piece of the testes or ovary constituted the gonad sample. A posterior section of the right lobe constituted the head kidney sample. The eye sample was the area of the eye surrounding the end of the *nervus opticus*. A section of the *medulla oblongata* constituted the brain sample. Homogenization of the tissue-samples was performed as described previously (26). Briefly, up to 40 mg of tissue were transferred to FastPrep Tubes containing SS metal beads lysing matrix (MP biomedicals) and lysisbuffer and homogenized in a FastPrep-24 5G homogenizator (MP biomedicals).

### Isolation of Leukocytes and *in vitro* Stimulation

Peripheral blood leukocytes (PBL) and head kidney leukocytes (HKL), were isolated from lumpfish (*n* = 8) using discontinuous Percoll gradients as described previously (27). A gentleMACS Dissociator (Miltenyi Biotec) was used to homogenize of the head kidney tissues. For the *in vitro* stimulation experiment, HKLs (4 × 10^7^ cells/well) were added to 24-well plates and stimulated for 18 h at 15°C with seven different ligands: 0.3 μg/ml triacylated lipopeptide (Pam3CSK4, Invivogen), 0.1 μg/ml diacylated lipopeptide (FSL-1, Invivogen), 20 μg/ml flagellin (FLA-BS, Invivogen), 50 μg/ml poly(I:C) (tlrl-pic, Invivogen), 10 μg/mg ssPoly (U)/LyoVec (Invivogen), 2 μM CpG (Eurogentec), or 2 μM GpC (Eurogentec). A leukocyte sample with medium added instead of ligands represented the non-stimulated control.

#### RNA Isolation and cDNA Synthesis

Total RNA was isolated from tissues and leukocytes using GenElute Mammalian Total RNA miniprep kit (Sigma) and treated with DNase I (Sigma) according to the manufacturer's instructions. A maximum of 2,500 ng RNA was used per 10 μl DNase reaction. To ensure that all traces of genomic DNA were removed from the samples and to validate the integrity and quality of the RNA, DNase treated RNA was assessed on a 1% agarose gel containing GelRed (Biotium) and quantified in a NanoDrop®ND 1000 UV-Vis spectrophotometer (NanoDrop Technologies). Subsequently, the RNA was reverse transcribed into cDNA using a qScript cDNA synthesis kit (Quanta Biosciences) according to the manufacturer's instructions using maximum 1,000 ng RNA per 20 μl reaction. The synthesized cDNA samples were stored at −20°C.

#### Quantitative PCR (qPCR)

qPCR was performed using a C1000 Touch Thermal Cycler with CFX96 Real-Time System (BioRad) using SYBR green JumpStart Taq Ready Mix kit for quantitative PCR (Sigma) and custom desalt primers from Sigma (see Tables 1 and 2). The PCR reaction contained 12.5 μl 2xSYBR Green JumpStart Taq Ready Mix, 10 μl cDNA (2 ng/μl for target genes and 0.2 ng/μl for reference genes), 1 μl (10 mM) forward and reverse primer and 0.5 μl nuclease and salt free water (Sigma). The reactions were thermo-cycled for 94°C at 5 min, followed by 40 cycles of 15 s at 94°C and 1 min at 60°C, until melt curve analysis were performed. Two-fold dilution curves (80–0.16 ng for the target and reference gene) were made for efficiency (E) calculations. Three parallel reactions were performed for all genes. Negative controls without template (NTC) and cDNA reactions without reverse transcriptase (-RT) were included for all master mixes. The –RT reaction ensured that the primers did not bind non-specifically.

**Table 1:** Primers used for qPCR, PCR, and Sanger sequencing.

**Gene**	**Accession numbers**	**Primer name**	**Sequence 5^**′**^-3^**′**^**	**Application**
RPS20		RPS20_F	GGAGAAGAGCCTGAAGGTGAAG	qPCR
		RPS20_R	GAGTTTTCCTGGTGGTGATGC	qPCR
IL-1β1	MN689238	IL-1β1_F	GACGGCGAGAAGCGGACCATAG	qPCR
		IL-1β1_R	TCAGGACAACTTTCTTGAGGTCAG	qPCR
IL-18	MN689239	IL-18_F	CCACCACAAGGCGCTGTTCTACA	qPCR
		IL-18_R	AGGCGGAGGACTCGAACTCGTA	qPCR
		IL-18intr_F1	CTGTTTCTTTCCAGAGTGCAAGTT	PCR and sanger seq.
		IL-18intr_R1	TCCACCTCGTCTTTGGCTTTTTC	PCR and sanger seq.
nIL-1F	MN689249	nIL-1F_F	CAAGTCCAACTGCTTCCTCCG	qPCR
		nIL-1F_R	ATCTTCTTCAACCTCTGCTTCTCG	qPCR
		nIL-1F_F353	ATGCAAAGCGGAAGCACAGACG	PCR and sanger seq.
		nIL-1F_R354	AAGTCTGAATGACGAAGAGGAACGATT	PCR and sanger seq.
		nIL-1F_F355	ACAGACGCACTGGGGGCTTTTA	Sanger seq.
		nIL-1F_R356	TGAATGACGAAGAGGAACGATTC	Sanger seq.
		nIL-1F_F357	ACAGTACTCAGTTTCAGCATGGAGATG	Sanger seq.
		nIL-1F_R358	ACCTCCTCTTGGAACGAGCATCACCT	Sanger seq.
		nIL_1F_R360	AACGAGCATCACCTCGCTGGATT	Sanger seq.
		nIL-1F_F422	CACATCCATCAGTGTCAGTGGCCTCTCAGTCCTAC	PCR and sanger seq.
IL-1β2	MN689240	IL-1β2_F	GAACATCAGCGACCACGAGGACAT	qPCR
		IL-1β 2_R	CAGGGACTCGAAGGTGTTCAGGGA	qPCR
		IL-1β 2intr_F156	ATGAGCGACTTTGATCTGTCTCAAGC	PCR and sanger seq.
		IL-1β 2intr_F362	TCGTCACGGCGACACAGAACT	PCR and Sanger seq.
		IL-1β 2intr_R363	ATCATAATGGAAACCTTCAAGCTGCACTAAA	PCR and sanger seq.

**Table 2:** qPCR assays.

**Target**	**Y**	***R*^**2**^**	**E**	**qPCR-product (bp)**
RPS20	−3.30	0.999	2.01	74
IL-1β1	−3.45	0.999	1.95	97
IL-18	−3.27	0.999	2.02	71
nIL-1F	−3.40	0.998	1.97	90
IL-1β2	−3.51	0.999	1.93	85

The gene expression in normal tissues were calculated by the ΔCq-method (Equation 1). The *in vitro* stimulation experiment was calculated by the ΔΔCq-method (Equation 2). All gene expression calculations utilized the housekeeping gene RPS20 as reference gene. Stability of the reference gene across tissues is shown in Supplemental Table 1.

$$\begin{array}{l} \text{Equation}1:\Delta Ct_{x}=\frac{{E_{target}}^{-\bar{x}}}{{E_{reference}}^{-\bar{x}}}\\ \text{Equation}2:\Delta \Delta Ct_{xy}=\frac{\frac{{E_{target}}^{-\bar{y}}}{{E_{reference}}^{-\bar{y}}}}{\frac{{E_{target}}^{-\bar{y}}}{{E_{reference}}^{-\bar{y}}}} \end{array}$$

#### Statistics

qPCR data were analyzed by two-way ANOVA in IBM® SPSS® Statistics (version 25.0.0.2) on log10 transformed data. The normal tissue data were followed up by Bonferroni corrected pairwise comparisons and the ligand stimulation data were followed up by Tukey's honest square difference *post-hoc* test. *F* values refers to the F statistic, df values refers to the degrees of freedom, *p*-values refer to the probability that the statistical summary of the population is equal or more extreme than the observed values of the sample, given that the null hypothesis is true (*p* < 0.05 is considered significant), and η^2^ refers to the effect size, or how much the relevant variable explains the observed variance.

### Sequence Identification and Database Mining

Individual transcripts in the transcriptome were annotated with BLAST matches, protein domains and GO terms using the Trinotate toolkit (https://github.com/Trinotate/Trinotate.github.io). The annotated transcriptome and differential gene expression (DEG)-data have been submitted to Array Express under accession number E-MTAB-6388.

Automatic annotated transcripts of IL-1β type II and nIL-1F in lumpfish were identified in a previous study (28). Further searches within the transcriptome using known sequences of IL-18, IL-1β type I, and IL-1 family receptors from other fish species, gave hits to several transcripts. Candidate sequences were identified by BLASTX against NCBI's non-redundant database and by phylogenetic analysis, which included all known full teleost sequences and swiss-prot entries for humans and mice. In order to perform multiple sequence alignment and phylogenetic analysis, sequences were mined from NCBI's protein database. Analysis of IL-1 family ligands was restricted to those from teleosts and the top 100 BLASTP hits using the lumpfish IL-1 family ligand sequences against NCBIs non-redundant database, in addition to sequences from (13) and (8). Replicate sequences, sequences <100 aa (amino acids) and >400 aa and severely deviating sequences were removed. This constituted a database of 273 teleost IL-1 family sequences, in addition to IL-1 and IL-18 sequences from humans and mouse (Supplemental Figure 5 and Supplemental Table 4).

#### Bioinformatic Analysis

Multiple sequence alignment was performed using MUSCLE (29) in UGENE (30). The phylogenetic maximum likelihood tree was constructed with IQ-TREE (31) using automatic model selection (32), followed by 100,000 bootstraps (33). An overview of species and gene identifier (GI) numbers included in the phylogenetic analysis are included in Supplemental Figure 5 and Supplemental Table 4. Domain predictions were performed using InterproScan (34). Transcriptome-wide DEG analyses of the signaling pathways NF-κB (KEGG map04064) and MAPK (KEGG map 04010) upon bacterial exposure were performed using data generated in Eggestøl et al. (28) and KEGG pathway analysis/KEGG Mapper tool as described previously (28). The synteny analyses were performed using Genomics (35). β-sheets in IL-1 β type I and IL-18 were identified using the human sequences as reference. The β-sheets in nIL-1F andIL-1β type II were predicted using BETApro Protein Beta Sheet Predictor (http://betapro.proteomics.ics.uci.edu/). Prediction of enzymatic cut sites in the lumpfish sequences were predicted using Peptide cutter (https://web.expasy.org/peptide_cutter/).

#### Gene Sequencing

The gene sequences of the four IL-1 family members were obtained from genome assembly (36) and/ or Sanger sequencing. A full-length IL-1 β type II gene was found in the assembled genome. The IL-18, nIL-1F, and IL-1β type I genes were PCR amplified from genomic DNA (isolated using Pure Core kit A, Quiagen, according to the manufacturer's instructions) using Phusion High-Fidelity DNA polymerase (Thermo scientific) and primers shown in Table 1. PCR products were purified by gel extraction (E.N.Z.A., Omega bio-tek) and sequenced at the DNA Sequencing Facility at the High Technology Centre in Bergen, Norway. The gene structure and exon-intron boundaries were determined by comparing transcripts from RNA sequencing of head kidney leukocytes from lumpfish (28) with scaffolds from genome assembly (36) and/or sequences obtained by Sanger sequencing.

#### Ethics Statement

The present work with lumpfish was conducted according to the approved national guidelines and performed according to prevailing animal welfare regulation. Rearing of fish under normal, optimal conditions does not require ethical approval under Norwegian law (FOR 1996-01-15 nr 23). All work in this manuscript has been done on tissues and cells harvested from dead fish. Fish were sacrificed with a sharp blow to the head, which is an appropriate procedure under Norwegian law.

## Results

### DEG Analysis of the NF-κ B—And MAPK Signaling Pathways

**Figure 1:**
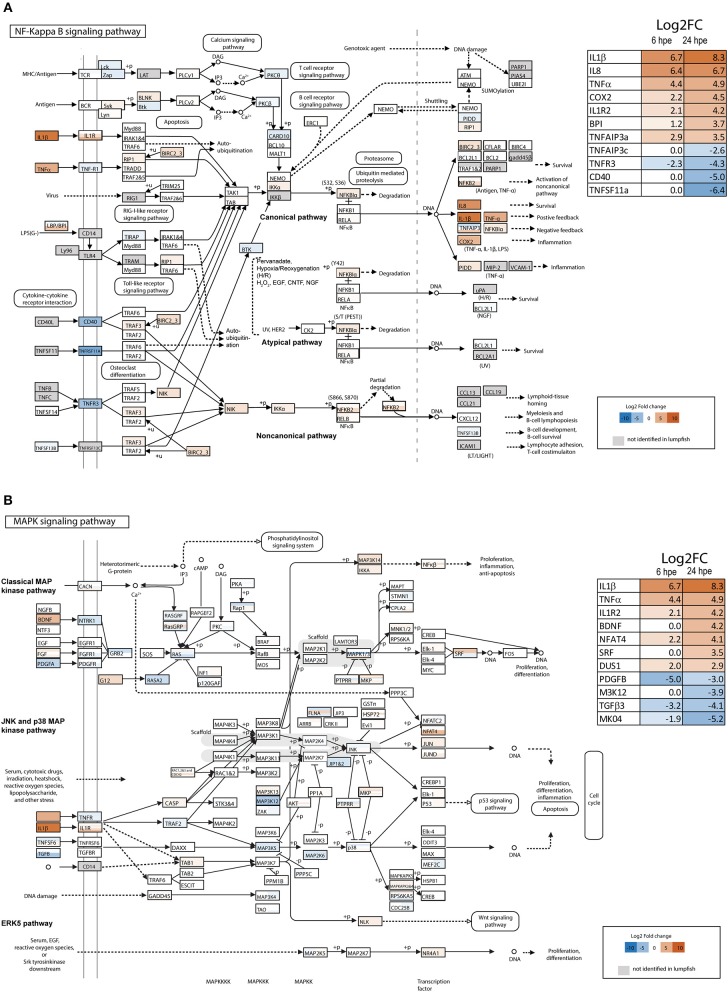
Overview of NF-κB and MAPK signaling pathway in lumpfish. **(A)** NF-κB signaling pathway (modified from KEGG map 04064) and **(B)** MAPK signaling pathway (modified from KEGG map 04010). The colors of the boxes refers to the respective gene's differential expression upon bacterial exposure and whether or not they are present in the lumpfish transcriptome. Gray—not present, a scale ranging from deep blue (very negative, log2 fold change = −10), through white (neutral, log2 fold change = 0), to brown-red (very positive, log2 fold change = 10) refers to the differentially expressed genes at 24 h. All genes that are identified in lumpfish, but not significant (adjusted *p* > 0.05) regulated is shown as white boxes. Differently regulated transcripts of the same gene are represented with horizontal bars within the respective gene-box. The 11 most regulated genes at 24 h are shown in the tables in the figure. See Supplemental Tables 2, 3 for a full list of DEG at 6 and 24 h post bacterial exposure.

IL-1β1 was the most significantly upregulated gene in lumpfish leukocytes 24 h post bacterial exposure (hpe) and was highly upregulated at both 6 and 24 hpe (34; Figure 1, Supplemental Tables 2, 3). To get further insight into the IL-1 signaling pathways, transcriptome-wide analysis of the NF-kappa B– and MAPK pathways were performed (Figures 1A,B). Most members of both pathways were identified in lumpfish (Figure 1, Supplemental Tables 2, 3). The DEG analysis upon bacterial exposure showed that the transcript level of genes belonging to the canonical NF-kappa B pathway (e.g., IL-1β1, IL-8, TNFα, and COX2) were most highly upregulated compared with the atypical and non-canonical pathway (Figure 1A and Supplemental Table 2), and the level of expression was higher at 24 hpe than 6 hpe (Figure 1A). Interestingly, IL1R2, the decoy receptor was among the most highly upregulated genes. Three TNFAIP3 transcripts were identified in the lumpfish transcriptome and these were regulated differently (Figure 1A, Supplemental Table 2), one (TNFAIP3a) being highly upregulated (Log2 fold 2.9 and 3.5 at 6 and 24 hpe, respectively), one (TNFAIP3c) strongly downregulated at 24 hpe (Log2 fold 0 and −2.6 at 6 and 24 hpe, respectively) and one (TNFAIP3b) that was not differentially regulated. In addition to TNFAIP3c, the transcripts that were most downregulated were the lymphotoxin beta receptor TNFR superfamily member 3 (TNFR3), Tumor necrosis factor receptor superfamily member 5 (CD40) and tumor necrosis factor receptor superfamily member 11A (TNFSF11a).

In the MAPK signaling pathway, transcription factors belonging to the classical MAP kinase pathway, JNK and p38 MAP kinase pathway and ERK5 pathway were upregulated. These included; brain-derived neurotrophic factor (BDNF), nuclear factor of activated T cells 3 (NFAT4), serum response factor (SRF) and Dual specificity Map kinase phosphatase (Figure 1B, Supplemental Table 3). Activation of these transcription factors leads to proliferation, differentiation and inflammation. The most downregulated transcripts in the MAPK signaling pathway were platelet-derived growth factor subunit B (PDGFB), transforming growth factor beta-3 (TGFB3) and the mitogen-activated kinases MAP3K12 and MK04.

### Identification and Molecular Characterization of IL-1 Family Ligands in Lumpfish

**Figure 2:**
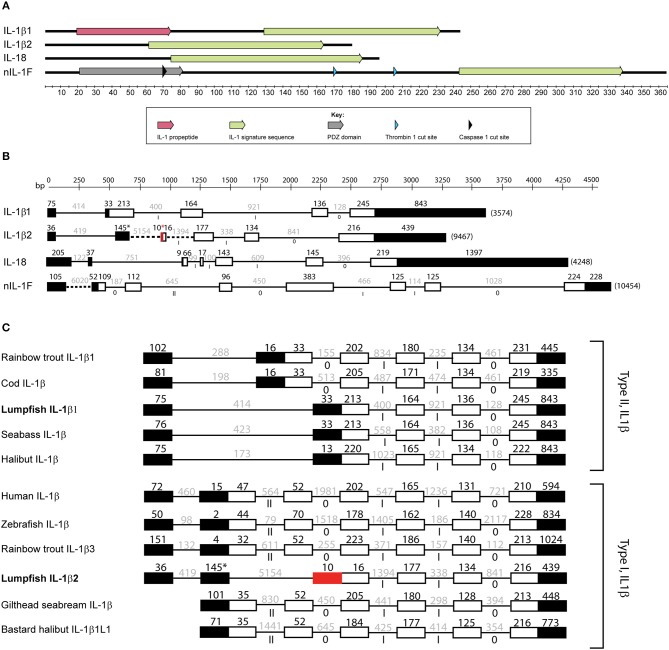
Molecular characterization of IL-1 ligands. **(A)** Domain and cut site prediction of IL-1β1, IL-1β2, IL-18, and nIL-1F. The x-axis is the number of amino acids from N-terminus to C-terminus of the proteins. IL-1 family domain is defined as IPR00097, IL-1 propeptide as IPR00302, and PDZ domain by IPR001478. **(B)** Exon/intron structure of the IL-1 ligands. Black boxes are non-coding exons, white boxes are coding exons. Red exon indicates remnants of the deleted exon in lumpfish IL-1β2. Thin lines are introns. Dotted lines are not to scale. **(C)** Non-coding exons (black boxes), coding exons (white boxes) for selected IL-1 β type I and type II sequences. Roman numerals represent intron phases.

In the previous transcriptome-wide analysis of lumpfish leukocytes, transcripts of IL-1β and a partial sequence of a new IL-1 family member (nIL-1F) were identified (28). To identify and get further insight into the ligands of the IL-1 family, sequences of known IL-1 family members from other teleost species were used as query sequences to search the lumpfish transcriptome. Using this approach, another transcript of nIL-1F was identified, as well as IL-1β2 and IL-18. All four family members contained the IL-1 family domain (Figure 2A). The IL-1β1 transcript consisted of 1,711 bp with a 759-bp open reading frame encoding a full-length protein of 252 aa (Figure 2A, Supplemental Figure 1). The deduced protein sequence contained an interleukin-1 propeptide (IPR00302) and the IL-1 family domain (IPR000975) (Figure 2A). IL-1β1 showed highest similarity to IL-1β of the sculpin *Trachidermus fasciatus* (Blast *E*-value 1E−142, 78% identity). An IL-18 candidate was identified. The transcript of 2,171 bp contained a 597-bp open reading frame encoding a 198 aa sequence that contained the IL-1 family domain and the IL-18 domain (IPR015529) (Figure 2A, Supplemental Figure 2). The blast hits with highest scores were five uncharacterized proteins and IL-18 from *Miichthys miiuy*. To obtain the full-length nIL-1F, two overlapping transcripts were merged. The sequence of the merged transcript, confirmed by Sanger sequencing, was 1,322 bp and contained an open reading frame of 1,059 bp encoding a 352 aa sequence. The start code was determined based on identification of a Kozak sequence (5′-G/ANNAUGG-3′). nIL-1F contained the IL-1 family domain and a PDZ domain (IPR001478) (Figure 2A, Supplemental Figure 3). The sequence showed highest similarity to an uncharacterized protein of *Notothenia coriiceps* (Blast *E*-value 0.0, 71% identity) and nIL-1F in *Gasterosteus aculeatus* (BLAST *E*-value 2E-158, identity 71%). The transcript encoding the IL-1β2 (1168 bp) contained an open reading frame of 543-bp encoding a 180 aa sequence. The lumpfish IL-1β2 sequence was shorter than IL-1β2 sequences in *Paralichthys olivaceus* and *G. aculeatus* (Supplemental Figure 4). A stop codon after the first predicted 11 amino acids was confirmed by Sanger sequencing. The sequence contained an IL-1 family domain (Figure 2A). The sequence showed highest similarity to an interleukin-1 receptor antagonist protein in *Larimichthys crocea* (*E*-value 1 < e-58, 52% identity). Caspase-1 and thrombin cut sites were only predicted in the nIL-1F sequence (Figure 2A).

### Gene Structure of the IL-1 Family Ligands in Lumpfish

Two types of IL-1β exist in fish, type I and type II, based on exon- intron structure. To determine the subtype of IL-1β in lumpfish, the gene sequence was analyzed. The IL-1β1 gene in lumpfish was 3,547 bp, contained five exon and four introns and is therefore a type II (Figure 2B, Supplemental Figure 6). IL-1β2 consisted of six exons and five introns. Interestingly, compared with other IL-1β2, the translated lumpfish sequence was much shorter (Figure 2B, Supplemental Figures 4, 9). A stop codon was present 33 bp downstream of the start codon followed by a deletion of 270 bp that corresponded to almost two whole exons (Supplemental Figure 4). The sequence upstream of the stop codon encoded MSDFDLSQALKR, which is similar to other IL-1β2 sequences (Supplemental Figure 4), showing that the stop codon and deletion is unique for lumpfish. Sanger sequencing confirmed the stop codon and deletion. Therefore, we predict that the lumpfish IL-1β2 consist of four coding exons. The first one is very short (MLQHD, due to the deletion) followed by three exons that are conserved in IL-1β2 sequences.

Comparison of gene structure of lumpfish the IL-1β sequences with other type I and type II sequences confirmed that lumpfish have both type I and type II IL-1β (Figure 2C). Also, the lumpfish sequences have the same intron phases as the other IL-1β sequences (Figure 2C). Due to deletion in the lumpfish IL-1β2 sequence, only the three exons in the C-terminal are similar to other sequences (Figure 2C, Supplemental Figure 4). Lumpfish IL-1β2 also have an extra exon N-terminal compared with other sequences, but that may be an artifact as this region consists exclusively of “g” and “t” (Supplemental Figure 4).

IL-18 had five coding exons and four introns, including the conserved short exon 2 and a predicted cut site (Figure 2B, Supplemental Figure 7). The nIL-1F gene consisted of coding seven exons and six introns (Figure 2B, Supplemental Figure 8).

### Synteny

Synteny analyses were performed for the IL-1 ligands. The locus of IL-1β1 in lumpfish was present on scaffold jcf7180000034562. IL-1β2 was present in scaffold jcf7180000030343. The 5' and 3'-end of the lumpfish IL-18 gene was present on two different scaffolds; jcf7180000029304 (position 1 to 1554) and jcf7180000030343 (position 1,542–4,248). Sanger sequencing was performed to obtain a full-length IL-18 gene, and the two scaffolds were combined. nIL-1F was found on scaffold 53, contig 235.

**Figure 3:**
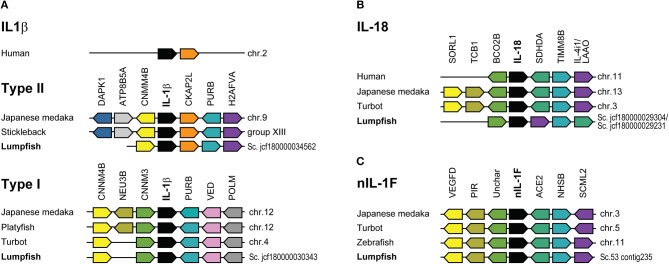
Synteny analysis of IL-1 ligands. **(A)** Human IL-1β locus and IL-1β type I and II in selected fish species. **(B)** IL-18. **(C)** nIL-1F. Schematic diagrams showing the genomic regions containing the IL-1 ligands. Lumpfish sequences are compared with Japanese medaka, stickleback, turbot, zebrafish, platyfish, and human. Conserved genes in the IL-1β and IL-18 locus in humans are shown for comparison. The IL-1 family ligands are shown in black boxes. Identical genes have same color.

There is a lack of well-characterized genomes from species closely related to lumpfish. In the comparative analysis, Japanese medaka, turbot, zebrafish, and human were chosen as they all provide well-characterized genomes of varying divergence time, approximately; 125, 125, 250, and 425 million years ago, respectively. The synteny of IL-1β1 was conserved among type II sequences and located in proximity to the genes death-associated protein kinase 1 (DAPK1), phospholipid-transporting ATPase (ATP8B5A), cyclin and CBS domain divalent metal cation transport mediator 4b (CNMM4B), cytoskeleton-associated protein 2-like (CKAP2L), purine-rich element binding protein Bb (PURB) and histone H2A (H2AFVA) (Figure 3A). In humans, the synteny of IL-1β is different from fish, and only IL-1β and CKAP2L are shared. Human IL-1β clusters together with IL-1α and other IL-1 family ligands. Lumpfish IL-1β2 possessed the same synteny as turbot, both lacking sialidase-3 (NEU3B) present in Japanese medaka (Figure 3). IL-1β type I and type II locus shear CNMM4B and PURB.

Synteny of the lumpfish IL-18 scaffolds, were similar to other species, except that succinate dehydrogenase complex, subunit D, integral membrane protein a (SDHDA) and l-amino acid oxidase (LAAO) have shifted position (Figure 3B). Further, the upstream context lacks tricalbin-1 (TCB1) and sortilin related receptor 1 (SORL1). Synteny analysis of nIL-1F was identical for all the species (Figure 3C).

### Phylogenetic Analysis of IL-1 Family Members

**Figure 4:**
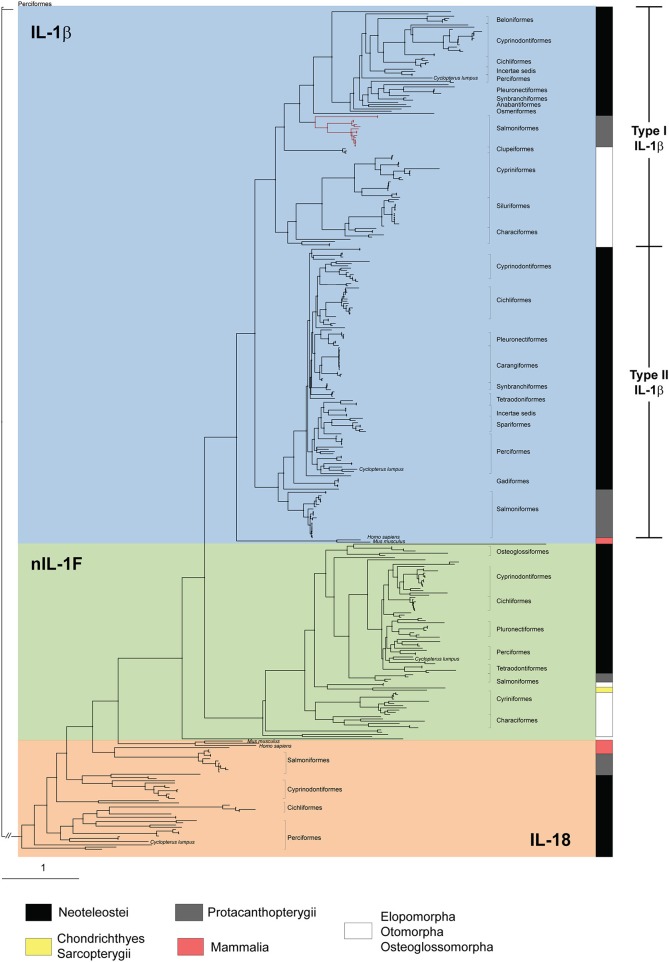
Phylogenetic tree of IL-1β type I and type II, IL-18, and nIL-1F. All full-length fish IL-1 ligands, and the human and mouse IL-1β and IL-18 sequences, are included. The phylogenetic tree was made using maximum likelihood as described in material and methods. Phylogenetic distance is indicated by branch length. Bar indicates distance of 1 substitution per amino acid site. Salmonid IL-1 β3 sequences within the type I group are shown with red line. A detailed phylogenetic tree which include sequence information (species and accession numbers) and bootstrap values (>80%) of 100,000 iterations is shown in Supplemental Figure 5 and listed in Supplemental Table 4.

To investigate the relationships among the IL-1 family ligands, a phylogenetic tree was constructed (Figure 4). All full-length teleost (taxid: 32443) IL-1 sequences available in NCBI, IL-1 ligand sequences described in the literature (8, 13), and human and mouse sequences (Supplemental Figures 4, 5) were included. In addition, as many of the fish sequences are as yet uncharacterized, we included all full-length teleost hits with adequate quality from a BLAST search using the lumpfish sequences as query sequences. The phylogenetic tree showed that nIL-1F and IL-1β, share a common ancestor (Figure 4). The nIL1F1 clade is a separate clade with sequences from all groups of fish, including cartilaginous (Chondrichthyes) and lobe-finned fish (Sarcopterygii). This suggest that nIL-1F, as IL-1β, may be an ancestral gene. IL-1β is divided into two subgroups; type II found in Neoteleostei and Protacanthopterygii, and type I found in species belonging to Elopomorpha, Otomorpha, and Osteoglossomorpha (EOO) as well as in species within Neoteleostei and Protacanthopterygii. The lumpfish sequences grouped within their expected group.

#### Expression Pattern of IL1β1, IL-1β2, IL-18, and nIL-1F in Tissues and Leukocytes

**Figure 5:**
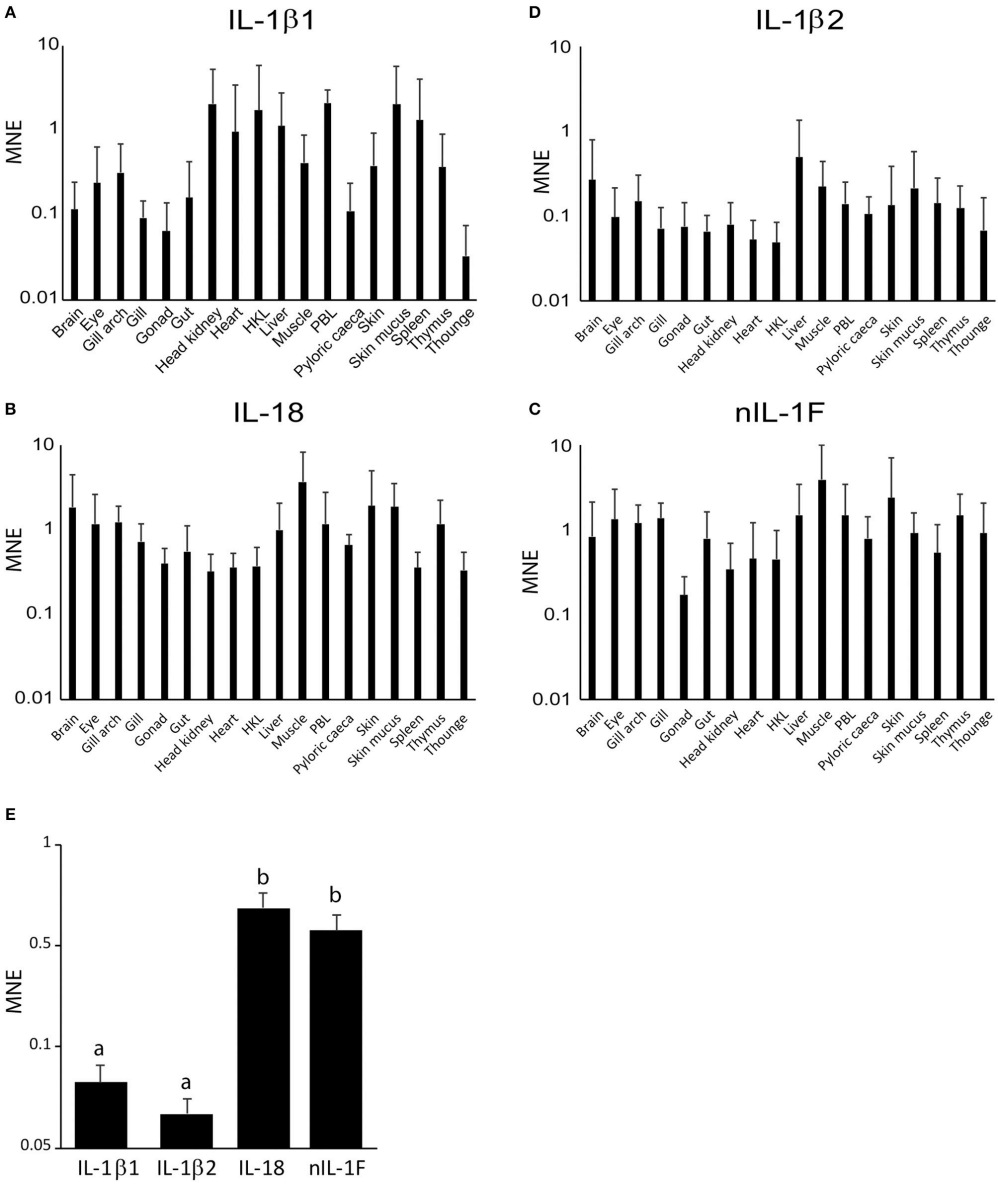
Tissue distribution of IL-1 ligand family members. Mean Normalized Expression (MNE) of the IL-1 ligands are shown relative to the reference gene (RPS20), plotted on a log10 scale. Error bars denote 1 standard deviation (SD). HKL, Head Kidney Leukocytes and PBL, Peripheral Blood Leukocytes. **(A)** IL-1β1. **(B)** IL18. **(C)** nIL-1F. **(D)** IL-1β2. **(E)** Tissue-independent expression of IL-1 ligands. Single letters = measurement that is statistically different the other.

mRNA transcript levels of the four IL-1 family ligands were measured by qPCR in sixteen tissues, as well as PBL and HKL. A two-way ANOVA analysis, investigating the effect of gene and tissue, showed that there was a significant effect of both gene [*F*_(3, 372)_ = 44.996, *p* = 7.8E-25, η^2^ = 0.266] and tissue [*F*_(17, 372)_ = 2.458, *p* = 0.001, η^2^ = 0.101]. An interaction effect was, however, not observed [*F*_(51, 372)_ = 0.547, *p* = 0.995, η^2^ = 0.070] between the factors, meaning that the genes were expressed in the different tissues, but at different levels. After examining the Bonferroni corrected *post-hoc* multiple comparisons and the column chart of the tissue-independent gene expression, it became apparent that the level of IL-18 and nIL-1F transcripts are statistically significantly higher than IL-1β1 and IL-1β2 transcripts (*p* < 0.001) throughout the tested tissues. IL-1β1 expression was highly variable, from an MNE value of 0.04 in tongue to more than 2.3 in PBL, skin mucus, head kidney, HKL and spleen (Figure 5A). The relative expression of IL-1β2 was generally low, except in the liver (Figure 5D). IL-18 and nIL-1F were abundant in most of the tissues analyzed (Figures 5B,C). High levels of IL-18 and nIL-1F transcripts were detected in skin and skin mucus, but surprisingly, also in muscle. The lowest detected level of nIL-1Fm was in the gonads. In contrast to IL-1β, the lowest values of IL-18 were in head kidney, HKL and spleen. Comparisons of the average transcript levels showed that the levels of IL-1β and IL-1β2 are statistically significantly lower than IL-18 and nIL-1F (Figure 5E).

#### Modulation of IL1β1, IL-1β2, nIL-1F, and IL-18 Expression in Head Kidney Leukocytes After Stimulation With Various Ligands *in vitro*

**Figure 6:**
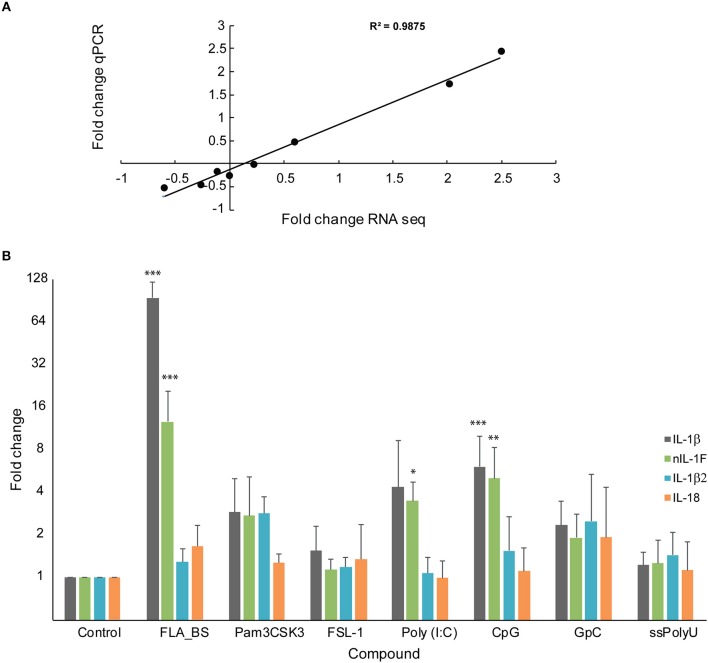
Gene expression of IL-1β, IL-18, nIL-1F and IL-1β2. **(A)** Correlation between log10 qPCR and RNA seq data of the IL-1 family members. **(B)** qPCR analyses of the IL-1 family members upon stimulation with various ligands. Error bars denote 1 standard deviation (SD). Stars denotes significant change compared with control (*P < 0.05, **P < 0.01, ***P < 0.001). The control is set to 1.

Transcriptome analyses of head kidney leukocytes upon bacterial exposure, showed that only IL-1β1 and, to a lesser extent, nIL1F, was upregulated [33, (Figure 1A)]. IL-18 was slightly, but significantly, downregulated (0.3-fold change) and IL-1β2 was not regulated. To compare the results from RNA sequencing (transcriptome data) with qPCR, we made cDNA of the RNA samples (*n* = 4) in the transcriptome analyses and performed qPCR. The results from RNA sequencing correlated well with qPCR analyses (*R*^2^ = 0.988) (Figure 6A). To further our understanding of the IL-1 family members' role in innate immunity, we stimulated head kidney leukocytes with seven different ligands (Figure 6B). IL-1β1 and nIL1F1 were most highly upregulated upon exposure to flagellin (FLA_BS), 100- and 12.7-fold change, respectively (*p* < 0.0001). These two genes were also highly upregulated upon exposure to poly (I:C) and CpG. IL-1β2 and IL-18 did not respond highly to any of the ligands, but they were both significantly upregulated upon exposure to Pam3CSK4 which is a synthetic triacylated lipopeptide (Figure 6B). The correlation analyses between the genes, showed that the expression levels of IL-1β1 and nIL1F are similar. IL18 and IL-1β2 are similar to each other, but different from the two aforementioned genes (Figure 6B). In summary, flagellin had the most highly significant effect on the HKL (*p* < 0.001), followed by CpG, Pam3CSK4 and poly (I:C) (Figure 6B). GpC, the diacylated lipoprotein FSL-1 and Poly(U) did not have a statistic significant effect on the expression levels of the IL-1 family members.

#### Identification of IL-Family Member Receptors

**Figure 7:**
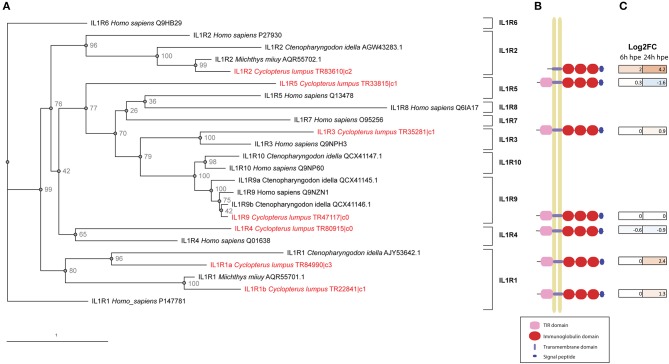
IL-1 receptors. **(A)** Phylogenetic tree including IL-1 receptors identified in lumpfish and in other species. Accession/trinity numbers of the sequences and bootstrap *p*-values are shown in the tree. **(B)** Schematic figure of full-length IL-1Rs identified in lumpfish transcriptome. The following domains are identified by interproscan: TIR domains (IPR035897) = pink, immunoglobulin domains (IPR007110) = red, transmembrane domain = light blue, signal peptide = dark blue. The IL1R3b sequence is not completely full-length, lacking about 10 amino acids in the N and C terminals. **(C)** DEG analysis of IL-1 receptors in lumpfish upon bacterial exposure. The color of the boxes reflects the DEG-value. Brown = upregulated, blue = downregulated.

In the lumpfish transcriptome, full-length sequences of the receptors IL-1R1 (two transcripts), IL1R2, IL1R3 (also known as IL-1RAcP), IL-1R4 (also known as ST2, which bind IL-33), IL-1R5 (also known as IL-18Rα) IL-1R9 (also known as TIGIRR-2) and DIGIRR were identified (Table 3, Figure 7). Both IL-1R1 transcripts were significantly upregulated at 24 h post bacterial exposure. IL-1R2 was also significantly upregulated at both times points. Interestingly, both IL-1R4 and IL-1R5 were down regulated. As shown in Figure 7, the full-length IL-1R1s, IL-1R3, IL-1R4, IL-1R5, and IL-1R9 have a signal peptide, three immunoglobulin domains, a transmembrane region and an intracellular domain. IL-1R2 is similar, but lacks the intracellular domain needed for downstream signaling. Furthermore, a lumpfish transcript showing high similarity to the C-terminal of fish specific IL-1R like family member double Ig-1R related molecule (DIGIRR) was identified (Table 3), having the conserved amino acid sequences ISRSRRLIV and FWKELALAMP, similar to other described DIGIRR sequences (35). IL-18R2 and IL-36R were searched for using known sequences from teleosts but were not found in the transcriptome.

**Table 3:** Overview of the IL1-family receptors identified in lumpfish.

Name^*^	**Alias**	**Accession numbers**	**Lumpfish Gene-ID**	**BLAST hit gene**	**BLAST hit specie**	***E*-value**	**Acc. no**
IL-1R1	IL-1R1a	MN689241	TR84990|c3	Interleukin-1 receptor type 1-like isoform X1	*Monopterus albus*	0	XP_020469769.1
	IL-1R1b	MN689242	TR22841|c1	Interleukin-1 receptor type 1-like isoform X2	*Lates calcarifer*	0	XP_018551896.1
IL-1R2	IL-1R2	MN689243	TR83610|c2	IL-1RII	*Miichthys miiuy*	8.00E-143	AQR55702.1
IL-1R3	IL-1RAcP/IL-1RAP	MN689244	TR35281|c1	Interleukin-1 receptor accessory protein-like 1 isoX3	*Monopterus albus*	0	XP_020447933.1
IL-1R4	ST2 /IL33R	MN689246	TR80915|c0	Interleukin-1 receptor-like 1	*Larimichthys crocea*	0	XP_027147138.1
IL-1R5	IL-18Rα	MN689247	TR33815|c1	PREDICTED: interleukin-18 receptor 1-like	*Lates calcarifer*	0	XP_018539216.1
IL-1R9	TIGIRR-2	MN689245	TR47117|c0	Interleukin-1 receptor accessory protein-like iso X1	*Mastacembelus armatus*	2.00E-177	XP_026183142.1
DIGIRR	DIGIRR	MN689248	TR66708|c0	Double immunoglobulin IL-1R-related protein	*Gasterosteus aculeatus*	1.00E-132	ACA51853.1

* *Same nomenclature as Boraschi et al. (18)*.

## Discussion

In a previous transcriptome-wide study of lumpfish leukocytes, we identified IL-1β1 and a partial sequence of a new interleukin-1 family member, nIL-1F. Both were upregulated upon bacterial exposure (28). To further our understanding of the biological processes involving IL-1β, we characterized the NF-kB and MAPK pathways by identifying signaling components and performed differential gene expression analyses upon bacterial exposure. Most components of the signaling pathways were found in the lumpfish transcriptome. In addition to the pro-inflammatory cytokines (IL-1 β, TNF-α and COX-2) which were highly upregulated, two of the most highly upregulated genes were IKBA and IL-1R2, both involved in regulation of IL-1β. IL-1β is regulated both at the transcriptional—and protein level where antagonists, IL-1Ra in mammals and nIL-1F in fish, bind to the IL-1R1 and block downstream signaling (14). There are also so-called decoy receptors, lacking an intracellular signaling domain that bind to IL-1 and prevent downstream signaling. Knowledge of the underlying mechanisms for regulation and signaling pathway is important as dysregulation can lead to acute and chronic inflammatory conditions (20, 25, 37).

Mammalian species have 11 ligands belonging to the IL-1 family. To date, only two of these are identified in fish; IL-1β and IL-18 (7). Fish have, however, one member that are unique for fish; the nIL-1F. In the current study, we have identified and characterized full-length sequences of IL-1β1 (type II), IL-1β2 (type I), IL-18 and nIL-1F. Analysis of their predicted protein sequences showed that they all possess the IL-1 signature sequence. Interestingly, the lumpfish IL-1β2 is shorter than other teleost sequences. After the 12 first amino acids, a stop codon is present, and it lacks 69 amino acids N-terminally compared with Japanese flounder (Supplemental Figure 4). The rest of the lumpfish IL-1β2 sequence, which encodes the IL-1 signature and the characteristic β-strands that form a beta barrel, is present and shows similarity to other IL-1β type I described in other teleosts (8).

Gene expression analyses of the four IL-1 family members in different tissues and leukocytes from peripheral blood and head kidney in lumpfish showed that IL-18 is constitutively expressed in all tested organs. This is similar to other studies [reviewed in (7)]. Upon stimulation of head kidney leukocytes with various PAMPs, there were no significant difference in expression levels of lumpfish IL-18. Lack of differential IL-18 gene expression upon stimulation *in vitro* with PAMPs, recombinant proteins or pathogens has also been reported for other species such as trout (10) and sea bream (38). In chicken, IL-18 is a major growth factor for CD4+ T-cells and can stimulate their IFN-γ production. Use of IL-18 as an adjuvant in anti-viral vaccines has shown promising results (2). IL-18 is involved in both T cell type 1 and type 2 responses (11). Much less is known about the function and regulation of IL-18 in fish, but it has been suggested that alternative splicing might regulate IL-18 activity (2). IL-18BP in human is a secreted protein with high affinity for IL-18. IL-18BP is involved in down-regulation of Th1 responses, as well as controlling Th2 cytokine responses [reviewed in (25)]. IL-18BP candidates from different species of fish are present in public databases, but these have not yet been characterized functionally.

Our results show that nIL-1F, like IL-18, was highly expressed in most organs. Upon exposure to various PAMPs, the expression pattern to nIL-1F was similar to IL1β1, being most highly upregulated upon stimulation with flagellin and CpG. Studies from other species have shown that nIL-1F inhibits expression of IL-1β1 rather than initiates expression of pro-inflammatory cytokines, suggesting it is an antagonist of IL-1β1 (13). Antagonistic effect of nIL-1F has also been demonstrated in grass carp where it binds to IL-1β receptor type 1 and attenuates IL-1β activity in HKL (14). IL-1β2 was expressed at low levels in most studied organs in lumpfish and stimulation with different PAMPs did not cause significant differences in expression levels. Weak induction of IL-1β type I upon PAMP activation has also been shown in gilthead seabream (8) and Japanese flounder (12). The function of IL-1β type I is not fully understood, but it plays a role in innate immunity activating respiratory burst activity of phagocytes (8). Down-stream signaling of IL-1β type I has not yet been investigated, but crosstalk between Toll-like receptors (TLRs) and IL-1β type I has been suggested by (8).

**Figure 8:**
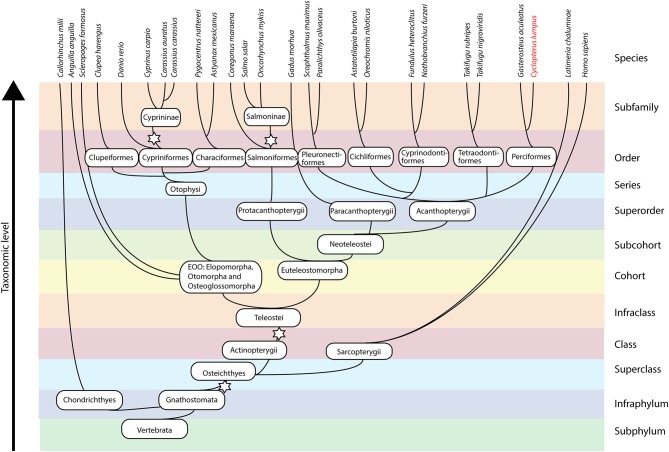
Simplified phylogeny of fishes including selected taxonomic units. The stars denote whole genome duplication events.

Our phylogenetic analysis confirms that lumpfish possess all IL-l ligands. IL-1β type I sequences in species belonging to Neoteleostei are possibly paralogs of a last common ancestor (LCA) leading to IL-1β3 in salmoniform fish, as it is the sister group to the IL-1β type I clade in the most evolutionary advanced fishes, and a case of neofunctionalization leading to the creation of a new gene. The phylogenetic tree clearly shows the separation of IL-1β into two groups; type I and type II. Separation into two groups is further supported by the exon-intron structure and intron phases (Figure 2C). The synteny analysis that shows similarity between the IL-1β type I and II loci, e.g., proximity to CNNM4 and PURB (Figure 3). The origin of the two IL-1 β types was likely the third round of the teleost-specific whole genome duplication (3R WGD) (Figure 8). Further, the nIL1-F1 clade contains members from Sarcopterygii *(Latimeria chalumnae)* and Chondrichthyes *(Callorhinchus milii)* and is placed between the IL-1β and IL-18 clades, indicating that this is an old gene that stems back to before the spilt of Eugnathostomata (~430 mya) and the development of the adaptive immune system. It is likely that the tetrapod lineage has lost the nIL-1F gene somewhere along its evolution. Interestingly, the Neoteleostei clade is placed closer to the Euteleostomorpha clade than the Elopomorpha/Otomorpha/Osteoglossomorpha clade, displaying that the Otomorpha clade is evolutionarily more distant to most teleosts than the ghost shark and lobe-finned fishes, in terms of nIL-1F homology. It is therefore likely that Otomorpha nIL-1F gene possesses traits that are not necessarily consistent with other teleost nIL-1F traits.

In the current study, we have also identified six IL-1R like receptors; IL-1R1 (two transcripts), IL-1R2, IL-1R3, IL-1R4, IL-1R5, and IL-1R9 in addition to a double Ig IL-1R related molecule (DIGIRR). Differential gene expression analyses of lumpfish HKL exposed to *V. anguillarum*, showed that both transcripts of IL1R1, IL1R2, and IL-1R3 were significantly upregulated. Of these, IL1R2, which is a negative regulator for the IL-1 system, was most highly upregulated (18-fold upregulated). It is of major importance to balance amplification of innate immunity and uncontrolled inflammation that can lead to diseases [reviewed in (20, 39)]. In mammals, IL1R2 negatively regulates IL-1 activity by different mechanisms [reviewed in (40)]. In short, it can act as a decoy receptor for IL-1 (both IL-1 α and IL-1 β), a dominant –negative molecule and scavenger. Also, IL1R2 can bind to proIL-1α and proIL-1 β in cytoplasm and thus avoid processing by caspase-1 and thus activation. Another negative regulator of the IL-1 signaling in mammals is the receptor called single Ig IL-1R related molecule (SIGIRR, also known as TIR8/ IL-1R8). SIGIRR is also present in ancient vertebrates such as zebrafish (41). However, some fish such as green spotted puffer, Japanese puffer and three-spined stickleback do not have SIGIRR, but have instead a related receptor with two Ig domains called double Ig IL-1R related molecule (DIGIRR) which is a negative regulator of IL-1 signaling similar to SIGIRR (42). In lumpfish, we found a DIGIRR candidate with two Ig domains and the conserved amino acids A L similar to other DIGIRR sequences. An understanding of how production of cytokines is regulated, through their receptors and signaling pathways, gives the potential to modulate their activity, e.g., through immune stimulation.

## Conclusion

In the current study, we have identified and characterized members of the IL-1 family of cytokines, as well as their receptors and down-stream signaling pathways. Our data constitutes an important foundation for further elucidation of cytokine functions, protein-protein interactions and the underlying mechanisms for regulation of these molecules in lumpfish and provide in-depth insight into the phylogeny of the IL-1 ligand family. In-depth knowledge of the innate and adaptive immunity will contribute to increased welfare of lumpfish as it forms the basis for development for immune prophylactic measures. Furthermore, genomic and transcriptomic data of lumpfish is also of interest as it represents a phylogenetic group (Cottales) that is usually not included in comparative and phylogenetic analyses.

## Data Availability Statement

The accession numbers for data in this study are included in the article. Other raw data supporting the conclusions of this article will be made available by the authors, without undue reservation, to any qualified researcher.

## Ethics Statement

Ethical review and approval was not required for the animal study because the animals used in this research was not subject to any experimental procedure, as all experiments was conducted *in vitro* on harvested samples.

## Author Contributions

HE, HL, and GH performed analysis. TK provided genomic sequences. HE and GH wrote the initial draft. All co-authors contributed to proofreading and editing the manuscript.

### Conflict of Interest

TK is employed by the company Aquagen AS. The remaining authors declare that the research was conducted in the absence of any commercial or financial relationships that could be construed as a potential conflict of interest.

## Supplementary Material

Supplemental Figure 1 IL1β alignment. Alignment of IL-1β sequences from human, mouse and teleosts, including the lumpfish sequence. Underlined amino acids are members of the IL-1 family signature. The purple horizontal line indicates the mammalian caspase 1 cut site. β-sheets are indicated by gray boxes.

Supplemental Figure 2 IL18 alignment. Alignment of IL-18 sequences from human, mouse and teleosts, including the lumpfish sequence. Underlined amino acids are members of the IL-1 family signature. The first purple horizontal line indicates a teleost cut site identified in (10). The second purple horizontal line indicates the mammalian caspase 1 or 4 cut site. Regions that contain β-sheets are indicated by gray boxes.

Supplemental Figure 3 nIL-1F alignment. Alignment of teleost, including lumpfish, IL-1β sequences. Underlined amino acids are members of the IL-1 family signature. The purple horizontal line indicates the caspase 1 cut site predicted in the lumpfish sequence. The blue horizontal lines indicate the thrombin cut sites predicted in the lumpfish sequence. β -sheets are indicated by gray boxes.

Supplemental Figure 4 IL-1β type I alignment. Alignment of teleost sequences, including lumpfish IL-1β2. Underlined amino acids are members of the IL-1 family signature. β-sheets are indicated by gray boxes.

Supplemental Figure 5 Phylogenetic tree of IL-1 ligands with accession numbers. The colors of the clades are similar to Figure 4. Bootstrap values > 80 % (of 100,000 iterations) are shown in the tree. The lumpfish sequences are written with red letters.

Supplemental Figure 6 Nucleotide and deduced amino acid sequences for lumpfish IL-1β. gDNA specific sequence is represented with lower case, cDNA sequence with upper case, and cDNA sequence is represented with bold letter. Intron-exon boundaries (gt and ag) are encircled.

Supplemental Figure 7 Nucleotide and deduced amino acid sequences for lumpfish IL-18. gDNA specific sequence is represented with lower case, cDNA sequence with upper case and cDNA sequence is represented with bold letter. Intron-exon boundaries (gt and ag) are encircled.

Supplemental Figure 8 Nucleotide and deduced amino acid sequences for lumpfish nIL-1F. gDNA specific sequence is represented with lower case, cDNA sequence with upper case and cDNA sequence is represented with bold letter. Intron-exon boundaries (gt and ag) are encircle.

Supplemental Figure 9 Nucleotide and deduced amino acid sequences for lumpfish IL-1β2. gDNA specific sequence is represented with lower case, cDNA sequence with upper case and cDNA sequence is represented with bold letter. Intron-exon boundaries (gt and ag) are encircled.

Supplemental Table 1 Ct-values of RPS20 in selected tissues and leukocytes.

Supplemental Table 2 NFκB signaling pathway components identified in lumpfish, including DEG values upon bacterial exposure.

Supplemental Table 3 MAPK signaling pathway components identified in lumpfish, including DEG values upon bacterial exposure.

Supplemental Table 4 Sequences included in the phylogenetic tree.
