# Supplementary figures and images for: Interleukin-1 Ligands and Receptors in Lumpfish (Cyclopterus lumpus L.): Molecular Characterization, Phylogeny, Gene Expression, and Transcriptome Analyses
Source: Front Immunol. 2020 Apr 2;11:502. doi: 10.3389/fimmu.2020.00502 (PMC7144542; doi:10.3389/fimmu.2020.00502)

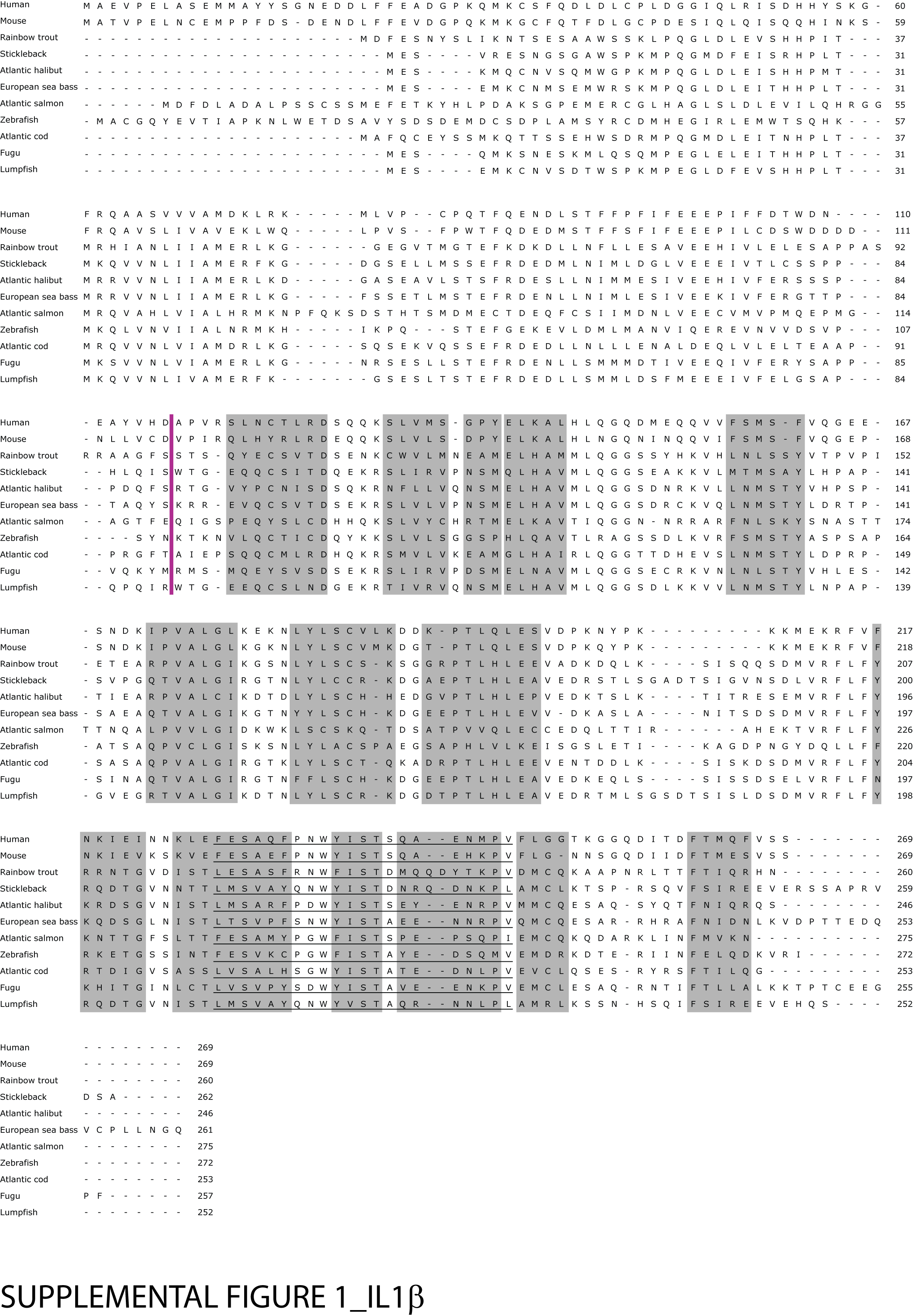

Supplement: Supplemental Figure 1 — IL1β alignment. Alignment of IL-1β sequences from human, mouse and teleosts, including the lumpfish sequence. Underlined amino acids are members of the IL-1 family signature. The purple horizontal line indicates the mammalian caspase 1 cut site. β-sheets are indicated by gray boxes. [file Image_1.TIF]

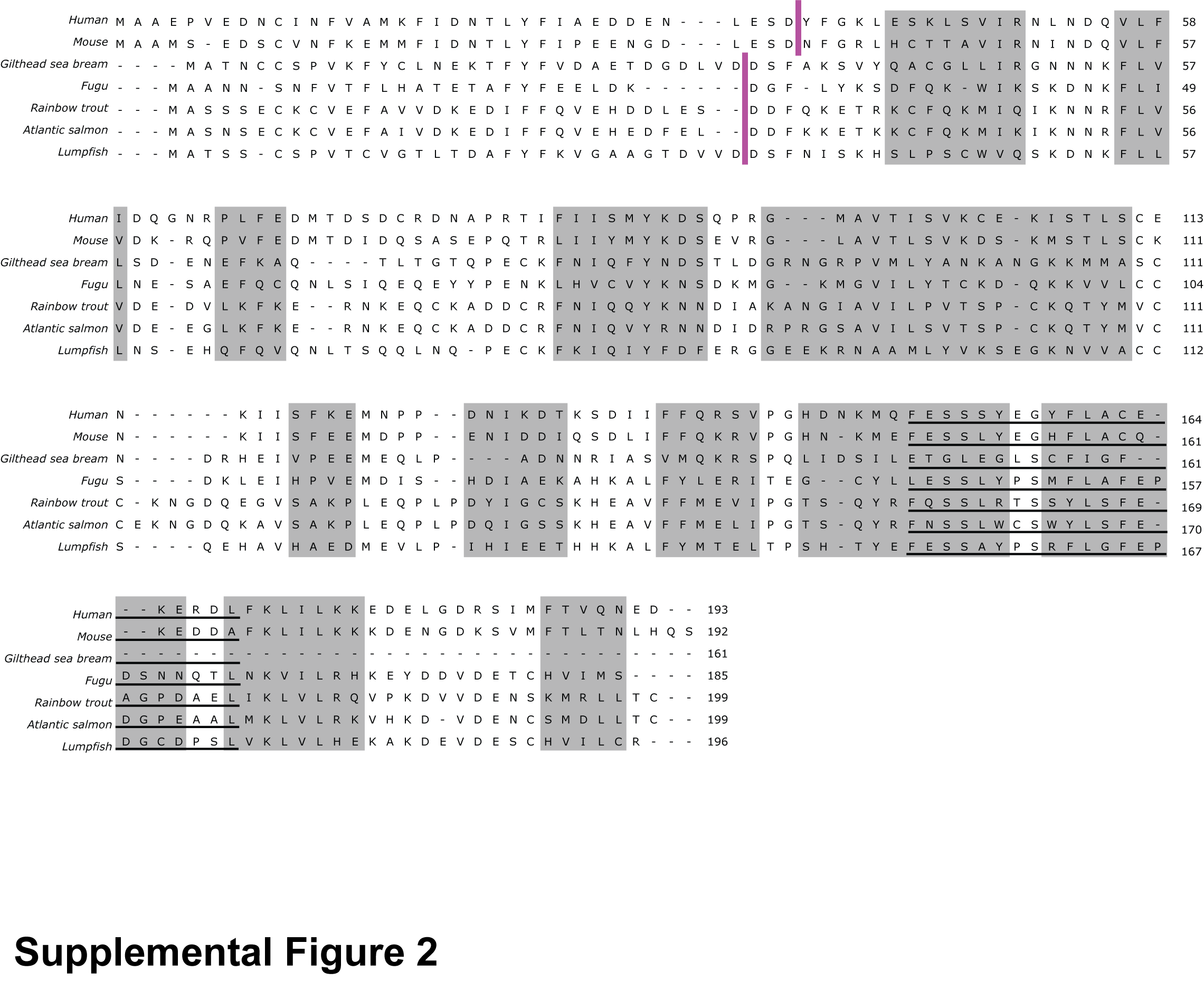

Supplement: Supplemental Figure 2 — IL18 alignment. Alignment of IL-18 sequences from human, mouse and teleosts, including the lumpfish sequence. Underlined amino acids are members of the IL-1 family signature. The first purple horizontal line indicates a teleost cut site identified in (10). The second purple horizontal line indicates the mammalian caspase 1 or 4 cut site. Regions that contain β-sheets are indicated by gray boxes. [file Image_2.TIF]

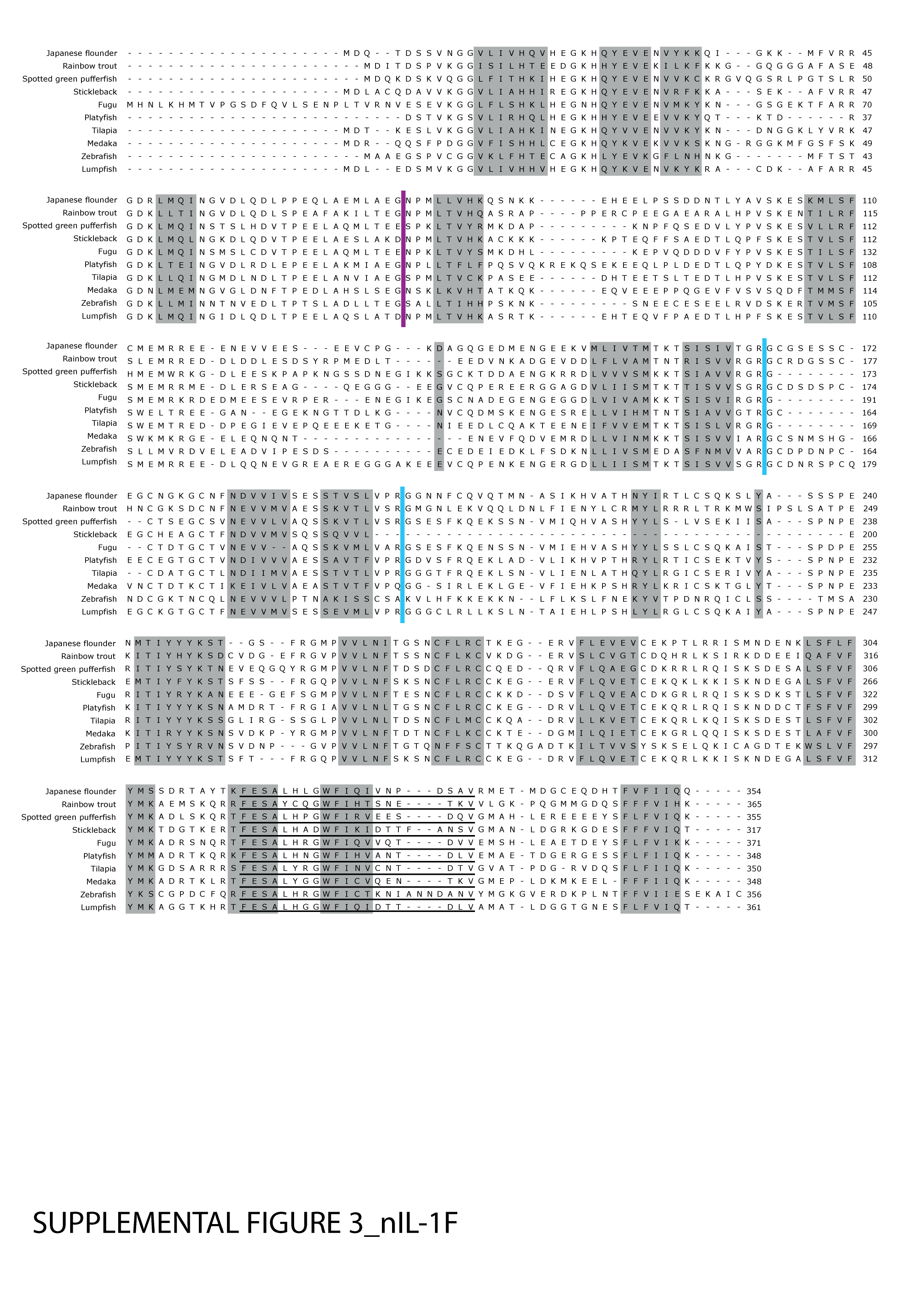

Supplement: Supplemental Figure 3 — nIL-1F alignment. Alignment of teleost, including lumpfish, IL-1β sequences. Underlined amino acids are members of the IL-1 family signature. The purple horizontal line indicates the caspase 1 cut site predicted in the lumpfish sequence. The blue horizontal lines indicate the thrombin cut sites predicted in the lumpfish sequence. β -sheets are indicated by gray boxes. [file Image_3.TIF]

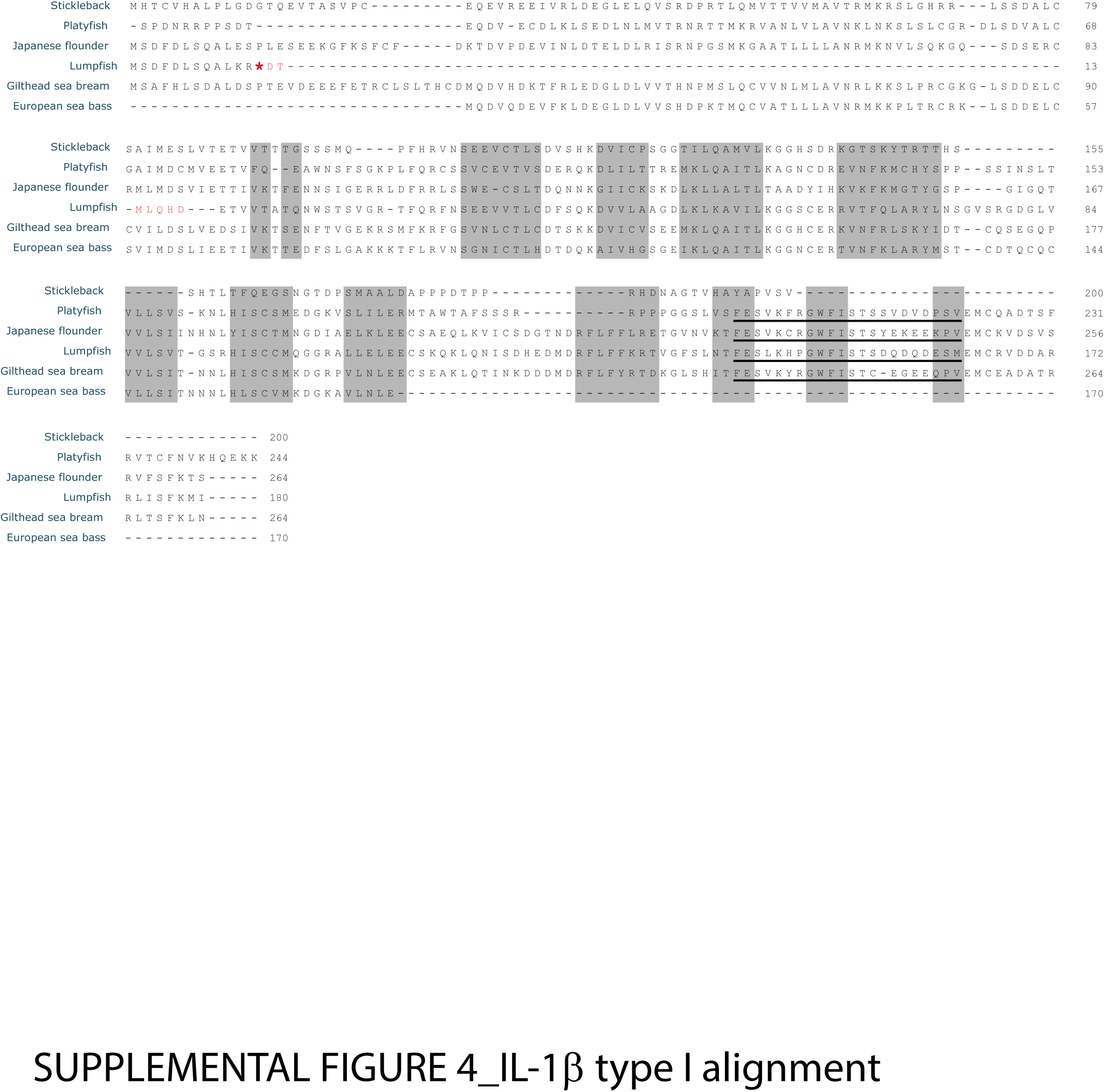

Supplement: Supplemental Figure 4 — IL-1β type I alignment. Alignment of teleost sequences, including lumpfish IL-1β2. Underlined amino acids are members of the IL-1 family signature. β-sheets are indicated by gray boxes. [file Image_4.TIF]

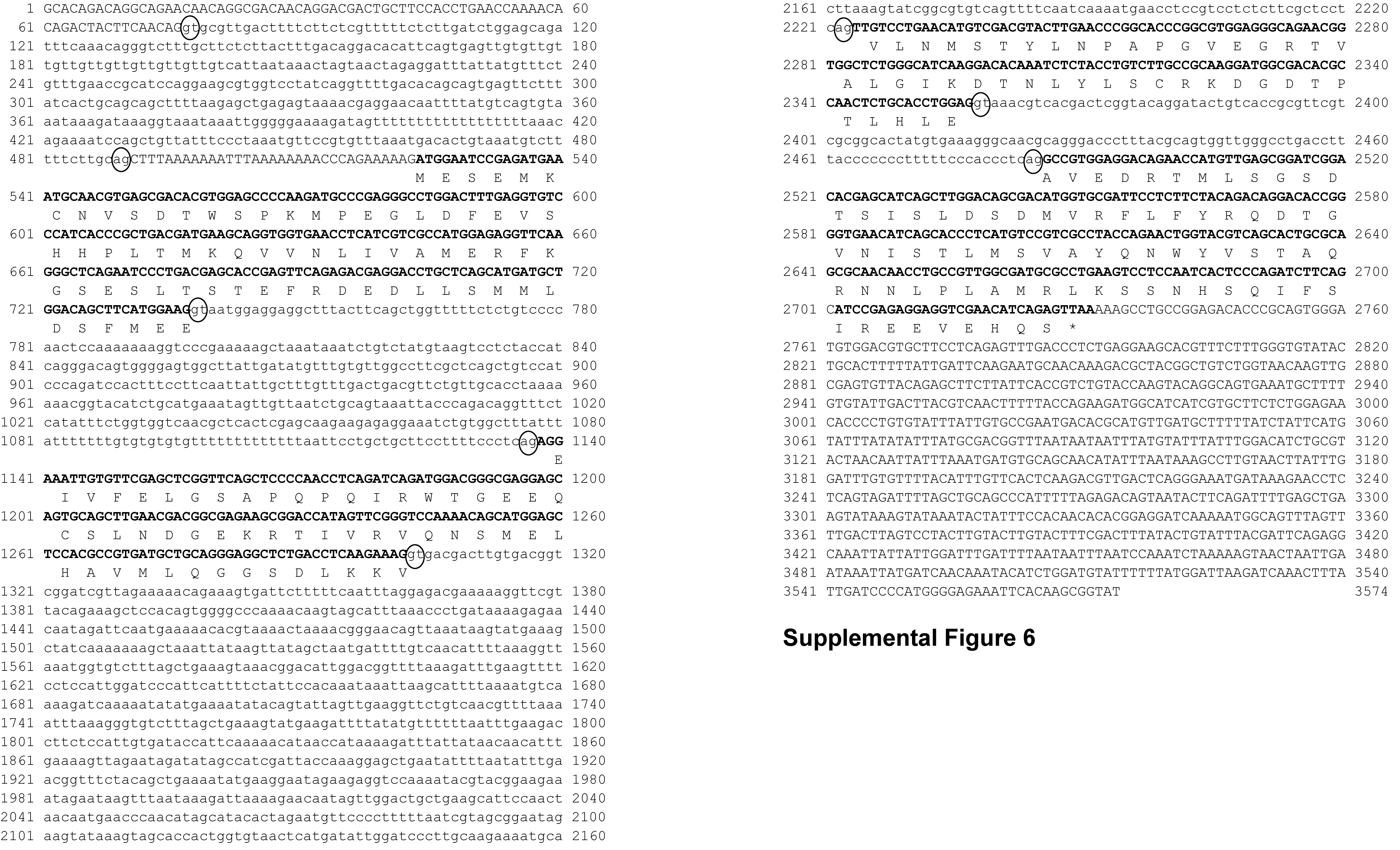

Supplement: Supplemental Figure 6 — Nucleotide and deduced amino acid sequences for lumpfish IL-1β. gDNA specific sequence is represented with lower case, cDNA sequence with upper case, and cDNA sequence is represented with bold letter. Intron-exon boundaries (gt and ag) are encircled. [file Image_6.tif]

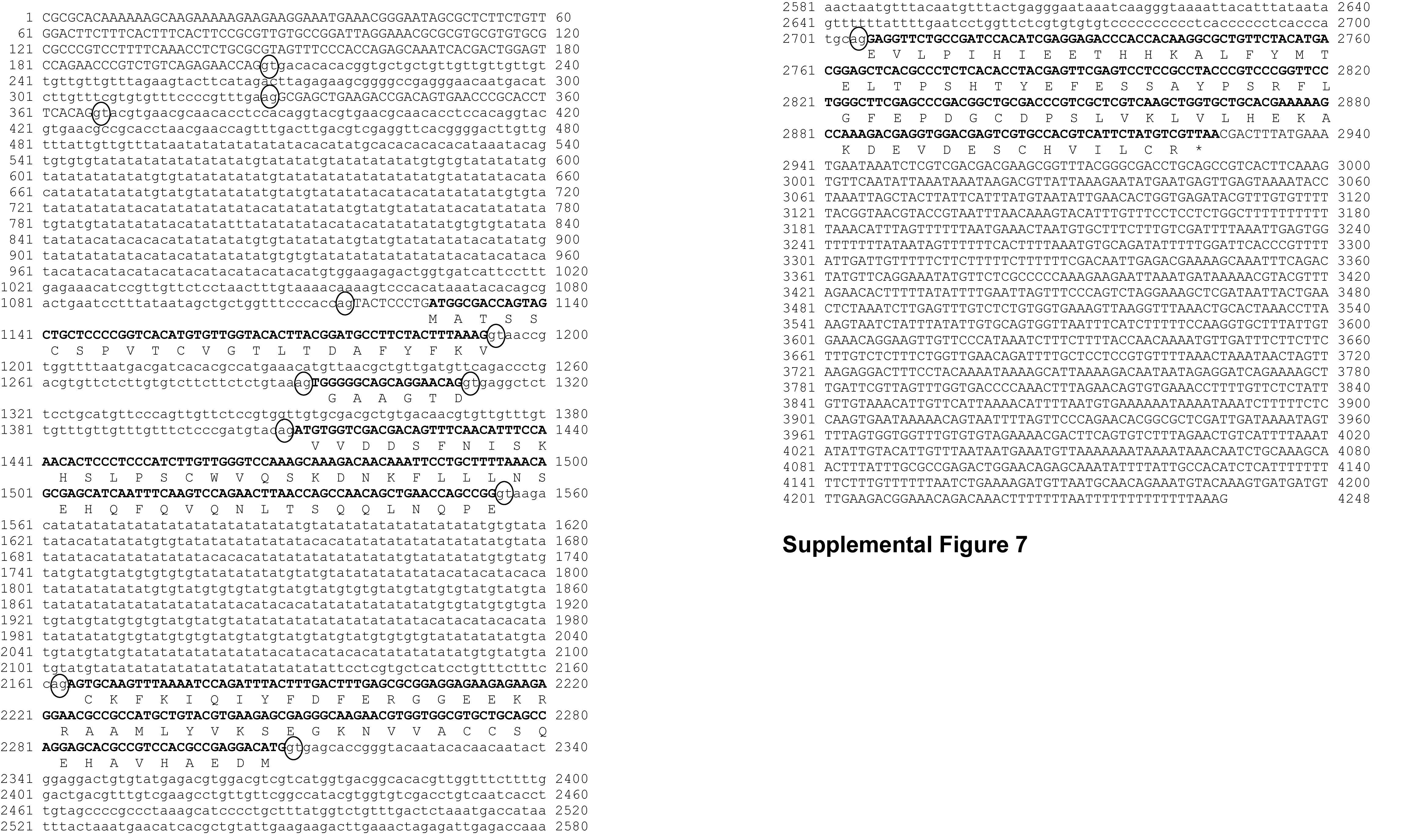

Supplement: Supplemental Figure 7 — Nucleotide and deduced amino acid sequences for lumpfish IL-18. gDNA specific sequence is represented with lower case, cDNA sequence with upper case and cDNA sequence is represented with bold letter. Intron-exon boundaries (gt and ag) are encircled. [file Image_7.tif]

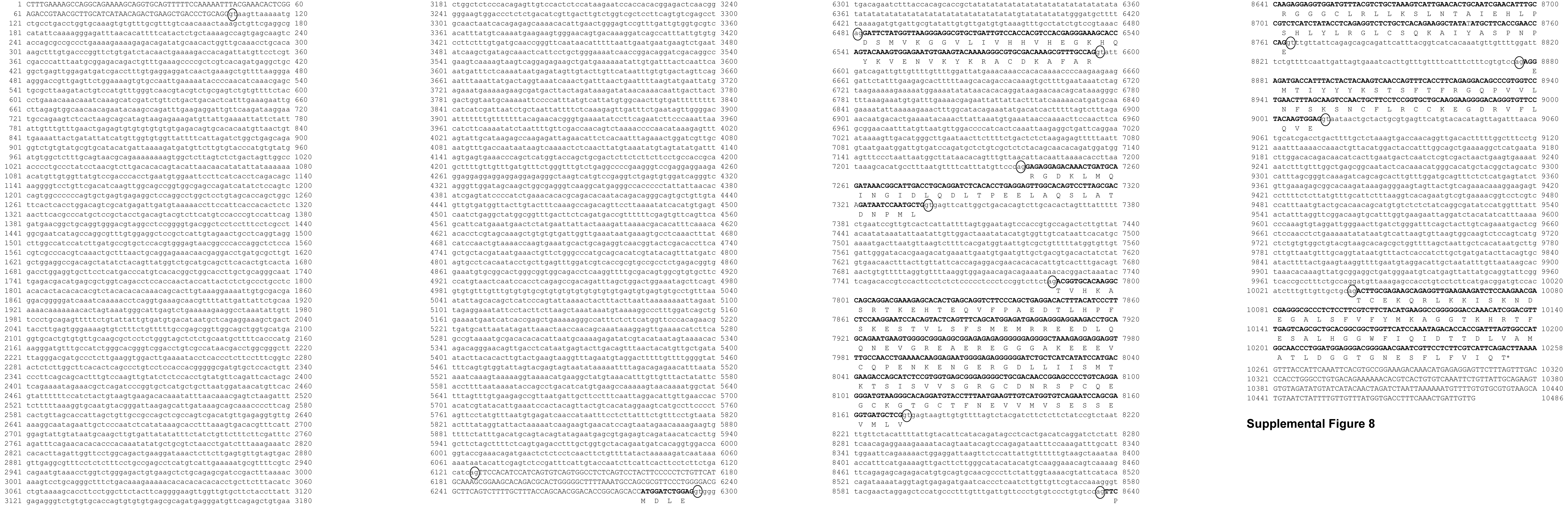

Supplement: Supplemental Figure 8 — Nucleotide and deduced amino acid sequences for lumpfish nIL-1F. gDNA specific sequence is represented with lower case, cDNA sequence with upper case and cDNA sequence is represented with bold letter. Intron-exon boundaries (gt and ag) are encircle. [file Image_8.tif]

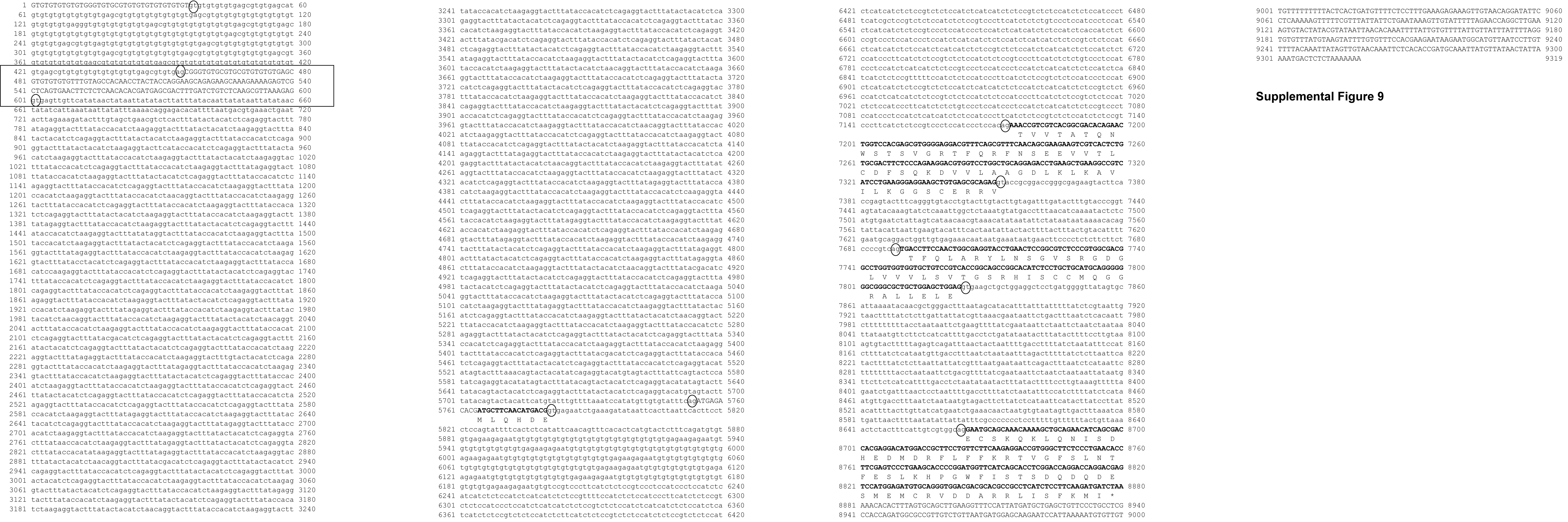

Supplement: Supplemental Figure 9 — Nucleotide and deduced amino acid sequences for lumpfish IL-1β2. gDNA specific sequence is represented with lower case, cDNA sequence with upper case and cDNA sequence is represented with bold letter. Intron-exon boundaries (gt and ag) are encircled. [file Image_9.tif]
