# Supplementary material for: Interleukin-1 Ligands and Receptors in Lumpfish (Cyclopterus lumpus L.): Molecular Characterization, Phylogeny, Gene Expression, and Transcriptome Analyses
Source: Front Immunol. 2020 Apr 2;11:502. doi: 10.3389/fimmu.2020.00502 (PMC7144542; doi:10.3389/fimmu.2020.00502)
Supplement: Supplemental Table 1 — Ct-values of RPS20 in selected tissues and leukocytes. [file Table_1.DOCX]

**Supplemental Table 1.** Ct-values of RPS20 in selected tissues and leukocytes.

| **Sample** | **Individual** | **Replicate** | **CT value** |
| --- | --- | --- | --- |
| HKL (non-stimulated) | 1 | 1 | 24.75 |
|  | 1 | 2 | 24.70 |
|  | 1 | 3 | 24.70 |
|  | 2 | 1 | 25.15 |
|  | 2 | 2 | 25.06 |
|  | 2 | 3 | 25.06 |
|  | 3 | 1 | 23.19 |
|  | 3 | 2 | 23.16 |
| HKL (stimulated with CpG) | 1 | 1 | 23.94 |
|  | 1 | 2 | 23.84 |
|  | 1 | 3 | 23.86 |
|  | 2 | 1 | 24.88 |
|  | 2 | 2 | 24.89 |
|  | 2 | 3 | 24.87 |
|  | 3 | 1 | 23.30 |
|  | 3 | 2 | 23.33 |
|  | 3 | 3 | 23.33 |
| Skin mucus | 1 | 1 | 26.71 |
|  | 1 | 2 | 26.86 |
|  | 1 | 3 | 26.75 |
|  | 2 | 1 | 26.50 |
|  | 2 | 2 | 26.54 |
|  | 2 | 3 | 26.32 |
|  | 3 | 1 | 26.50 |
|  | 3 | 2 | 26.64 |
|  | 3 | 3 | 26.40 |
| Head kidney | 1 | 1 | 25.19 |
|  | 1 | 2 | 25.40 |
|  | 1 | 3 | 25.36 |
|  | 2 | 1 | 25.50 |
|  | 2 | 2 | 25.73 |
|  | 2 | 3 | 25.75 |
|  | 3 | 1 | 26.16 |
|  | 3 | 2 | 26.19 |
|  | 3 | 3 | 26.12 |
| Brain | 1 | 1 | 26.83 |
|  | 1 | 2 | 27.01 |
|  | 1 | 3 | 26.98 |
|  | 2 | 1 | 26.95 |
|  | 2 | 2 | 27.09 |
|  | 2 | 3 | 26.99 |
|  | 3 | 1 | 26.42 |
|  | 3 | 2 | 26.44 |
|  | 3 | 3 | 26.38 |
