# Supplementary material for: Interleukin-1 Ligands and Receptors in Lumpfish (Cyclopterus lumpus L.): Molecular Characterization, Phylogeny, Gene Expression, and Transcriptome Analyses
Source: Front Immunol. 2020 Apr 2;11:502. doi: 10.3389/fimmu.2020.00502 (PMC7144542; doi:10.3389/fimmu.2020.00502)
Supplement: Supplemental Table 2 — NFκB signaling pathway components identified in lumpfish, including DEG values upon bacterial exposure. [file Table_2.docx]

**Supplemental Table 2.** NFκB signaling pathway components identified in lumpfish, including DEG values upon bacterial exposure.

| **Box name** | **Pathway name** | **Kegg annotation** | **Annotation** | **Log2 fold change 6h** | **Log2fold change 24h** |
| --- | --- | --- | --- | --- | --- |
| IL1β | Interleukin 1 beta | K04519 | IL1B | 6.7 | 8.3 |
| IL-8 | Interleukin-8 | K10030 | IL-8 | 6.4 | 6.7 |
| TNFα | Tumor necrosis factor superfamily. member 2 | K03156 | TNFA | 4.4 | 4.9 |
| COX2 | Prostaglandin-endoperoxide synthase 2 | K11987 | PGH2 | 2.2 | 4.5 |
| IL1R | Interleukin-1 receptor type 2 | K04387 | IL1R2 | 2.1 | 4.2 |
| LBP/BPI | Lipopolysaccharide-binding protein | K05399 | BPI | 1.2 | 3.7 |
| TNFAIP3a | Tumor necrosis factor. alpha-induced protein 3 | K11859 | T.P3 | 2.9 | 3.5 |
| NFκβ2 | Nuclear factor NF-kappa-B light polypeptide gene enhancer in B-cells 2 | K04469 | NFKB2 | 3.2 | 3.4 |
| IL1R1 | Interleukin 1 receptor type 1 | K04387 | IL1R1 | 0.0 | 2.4 |
| BIRC2_3 | Baculoviral IAP repeat-containing protein 2/3 | K16060 | BIR | 1.3 | 2.1 |
| BIRC2_3 | Baculoviral IAP repeat-containing protein 2/3 | K16060 | PIAP | 1.2 | 2.0 |
| NIK | Mitogen-activated protein kinase kinase kinase 14 | K04466 | M3K14 | 1.2 | 1.9 |
| CD40 | Tumor necrosis factor receptor superfamily member 5 | K03160 | CD40 | 2.1 | 1.7 |
| CCL4 | C-C motif chemokine 4 | K12964 | CCL4 | 1.6 | 1.7 |
| NFκBIα | NF-kappa-B inhibitor alpha | K04734 | IKBA | 2.6 | 1.5 |
| IL1R | Interleukin 1 receptor type 1 | K04386 | IL1R2 | 0.0 | 1.3 |
| TRAF3 | TNF receptor-associated factor 3 | K03174 | TRAF3 | 0.8 | 1.2 |
| IKKα | Inhibitor of nuclear factor kappa-B kinase subunit alpha | K04467 | IKKA | 0.0 | 1.2 |
| RIP1 | Receptor-interacting serine/threonine-protein kinase 1 | K02861 | RIPK1 | 0.0 | 1.2 |
| IRAK1&4 | Interleukin-1 receptor-associated kinase 1 | K04733 | IRAK4 | 0.4 | 1.1 |
| Syk | Spleen tyrosine kinase | K05855 | KSYK | -0.7 | 1.1 |
| TAB | TAK1-binding protein 1 | K04403 | TAB1 | 0.0 | 1.0 |
| IL1R | Interleukin 1 receptor type 1 | K04386 | IL1RAcP | 0.0 | 0.9 |
| ERC1 | ELKS/RAB6-interacting/CAST family member 1 | K16072 | RB6I2 | 0.3 | 0.8 |
| RELA | Transcription factor p65 | K04735 | REL | 0.5 | 0.8 |
| CFLAR | CASP8 and FADD-like apoptosis regulator | K04724 | CFLAR | 0.4 | 0.8 |
| RELA | Transcription factor p65 | K04735 | TF65 | 0.6 | 0.7 |
| TRIM25 | Tripartite motif-containing protein 25 | K10652 | TRIM8 | 0.0 | 0.7 |
| BCL10 | B-cell CLL/lymphoma 10 | K07368 | BCL10 | 0.0 | 0.6 |
| CK2 | Casein kinase II subunit beta | K03115 | CSK2B | 0.3 | 0.6 |
| RELB | Transcription factor RelB | K09253 | RELB | 0.9 | 0.5 |
| TRADD | Tumor necrosis factor receptor type 1-associated DEATH domain protein | K03171 | TRADD | 0.0 | 0.5 |
| PLCγ1 | Phosphatidylinositol phospholipase C. gamma-1 | K01116 | PLCG1 | 0.0 | 0.5 |
| TAB | TAK1-binding protein 3 | K12793 | TAB3 | 0.0 | 0.5 |
| Lyn | Tyrosine-protein kinase Lyn | K05854 | LYN | -0.6 | 0.3 |
| PLCγ2 | Phosphatidylinositol phospholipase C. gamma-2 | K05859 | PLCG2 | 0.0 | 0.3 |
| TNFAIP3b | Tumor necrosis factor. alpha-induced protein 3 | K11859 | ZRAN1 | 0.0 | 0.3 |
| CK2 | Casein kinase II subunit alpha | K03097 | CSK21 | 0.0 | 0.3 |
| CK2 | Casein kinase II subunit alpha | K03097 | CSK22 | 0.0 | 0.2 |
| NEMO | Inhibitor of nuclear factor kappa-B kinase subunit gamma | K07210 | SPS2 | 0.0 | 0.0 |
| MALT1 | Mucosa-associated lymphoid tissue lymphoma translocation protein 1 | K07369 | MALT1 | -0.4 | 0.0 |
| ATM | Serine-protein kinase ATM | K04728 | ATM | 0.0 | 0.0 |
| BCL2 | Apoptosis regulator Bcl-2 | K02161 | BCL2 | 0.0 | 0.0 |
| BCR | Immunoglobulin heavy chain | K06856 | HVM63 | 0.0 | 0.0 |
| BIRC4 | E3 ubiquitin-protein ligase XIAP | K04725 | XIAP | 2.4 | 0.0 |
| CXCL12 | C-X-C motif chemokine 12 | K10031 | SDF1 | 0.0 | 0.0 |
| IL1R | Interleukin 1 receptor type 1 | K04386 | IL1RAcP | 0.0 | 0.0 |
| NEMO | Inhibitor of nuclear factor kappa-B kinase subunit gamma | K07210 | OPTN | -0.4 | 0.0 |
| NFκB | Nuclear factor NF-kappa-B p105 subunit | K02580 | NFKB1 | 0.0 | 0.0 |
| NFκB2 | Nuclear factor NF-kappa-B light polypeptide gene enhancer in B-cells 2 | K04469 | DHE3 | 0.6 | 0.0 |
| TAB | TAK1-binding protein 2 | K04404 | TAB2 | 0.0 | 0.0 |
| TAK1 | Mitogen-activated protein kinase kinase kinase 7 | K04427 | M3K7 | -0.3 | 0.0 |
| TCR | T-cell receptor beta chain V region | K10785 | TVB4 | 0.0 | 0.0 |
| TNFSF14 | Tumor necrosis factor receptor superfamily member 14 | K05477 | TNF14 | 0.1 | 0.0 |
| TNFβ | Tumor necrosis factor superfamily. member 2 | K03156 | TNFB | 0.0 | 0.0 |
| TRAF5 | TNF receptor-associated factor 5 | K09849 | TRAF5 | 0.0 | 0.0 |
| TRIM25 | Tripartite motif-containing protein 25 | K10652 | STXA | 0.0 | 0.0 |
| TRAF6 | TNF receptor-associated factor 6 | K03175 | TRAF6 | 0.0 | 0.0 |
| BCR | Immunoglobulin heavy chain | K06856 | HV02 | 0.0 | 0.0 |
| UBE2I | Ubiquitin-conjugating enzyme E2 I | K10577 | UBC9 | -0.2 | 0.0 |
| BCR | Immunoglobulin heavy chain | K06856 | HV303 | -0.1 | -0.1 |
| ERC1 | ELKS/RAB6-interacting/CAST family member 1 | K16072 | ERC2 | 0.2 | -0.1 |
| TRAF2 | TNF receptor-associated factor 2 | K03173 | TRAF2 | -0.3 | -0.2 |
| BCL2L1 | Bcl-2-like 1 (apoptosis regulator Bcl-X) | K04570 | B2CL1 | -0.3 | -0.2 |
| TRIM25 | Tripartite motif-containing protein 25 | K10652 | TRI25 | -1.0 | -0.3 |
| TRIM25 | Tripartite motif-containing protein 25 | K10652 | TRI16 | -0.3 | -0.6 |
| IL8 | Interleukin 8-like | K10030 | IL8like | 1.4 | -0.6 |
| NFκB2 | Nuclear factor NF-kappa-B light polypeptide gene enhancer in B-cells 2 | K04469 | FA35A | 0.4 | -0.6 |
| TCR | T-cell receptor alpha chain V region | K10784 | TVA3 | 0.0 | -0.6 |
| PKCβ | classical protein kinase C beta type | K19662 | KPCB | 0.0 | -0.7 |
| TCR | T-cell receptor beta chain V region | K10785 | TRBC2 | 0.0 | -0.7 |
| LBP/BPI | Lipopolysaccharide-binding protein | K05399 | LBP | 0.0 | -0.8 |
| TIRAP | Toll-interleukin 1 receptor (TIR) domain-containing adaptor protein | K05403 | TIRAP | -1.2 | -0.9 |
| TNFSF13B | Tumor necrosis factor receptor superfamily member 13B | K05476 | TN13B | 0.0 | -0.9 |
| TRIM25 | Tripartite motif-containing protein 25 | K10652 | TRI29 | -0.8 | -1.0 |
| PIDD | Leucine-rich repeats and death domain-containing protein | K10130 | PIDD1 | -0.4 | -1.1 |
| TNF-R1 | Tumor necrosis factor receptor superfamily member 1A | K03158 | TNR1A | -2.5 | -1.1 |
| PKCθ | Novel protein kinase C theta type | K18052 | KPCT | 0.0 | -1.2 |
| Lck | Lymphocyte cell-specific protein tyrosine kinase | K05856 | BLK | 1.0 | -1.3 |
| TRIM25 | Tripartite motif-containing protein 25 | K10652 | TRI39 | 0.3 | -1.3 |
| ZAP | Tyrosine-protein kinase ZAP70 | K07360 | ZAP70 | 0.0 | -1.5 |
| Zap | Bruton agammaglobulinemia tyrosine kinase | K07370 | BTK | 0.0 | -1.5 |
| Lck | Lymphocyte cell-specific protein tyrosine kinase | K05856 | LCK | 0.0 | -1.6 |
| CARD10 | Caspase recruitment domain-containing protein 11 | K07367 | CAR11 | 0.0 | -1.7 |
| TNFAIP3c | Tumor necrosis factor. alpha-induced protein 3 | K11859 | OTU7A | 0.0 | -2.6 |
| TNFR3 | Lymphotoxin beta receptor TNFR superfamily member 3 | K03159 | TNFR3 | -2.3 | -4.3 |
| CD40 | Tumor necrosis factor receptor superfamily member 5 | K03160 | CD40 | 0.0 | -5.0 |
| TNFRSF11A | Tumor necrosis factor receptor superfamily member 11A | K05147 | TNR11 | 0.0 | -6.4 |
