# Supplementary material for: Interleukin-1 Ligands and Receptors in Lumpfish (Cyclopterus lumpus L.): Molecular Characterization, Phylogeny, Gene Expression, and Transcriptome Analyses
Source: Front Immunol. 2020 Apr 2;11:502. doi: 10.3389/fimmu.2020.00502 (PMC7144542; doi:10.3389/fimmu.2020.00502)
Supplement: Supplemental Table 3 — MAPK signaling pathway components identified in lumpfish, including DEG values upon bacterial exposure. [file Table_3.docx]

**Supplemental Table 3**. MAPK signaling pathway components identified in lumpfish, including DEG values upon bacterial exposure.

| **Box name** | **Pathway name** | **Kegg identifier** | **Annotation** | **LogFC 6hpe** | **LogFC 24hpe** |
| --- | --- | --- | --- | --- | --- |
| IL1β | Interleukin 1 beta | K04519 | IL1B | 6.7 | 8.3 |
| TNFα | Tumor necrosis factor | K03156 | TNFA | 4.4 | 4.9 |
| IL1R | Interleukin 2 receptor | K04387 | IL1R2 | 2.1 | 4.2 |
| BDNF | Brain-derived neurotrophic factor | K04355 | BDNF | 0.0 | 4.2 |
| NFAT4 | Nuclear factor of activated T-cells. cytoplasmic 3 | K17333 | NFAT5 | 2.2 | 4.1 |
| SRF | Serum response factor | K04378 | NA | 0.0 | 3.5 |
| MKP | Dual specificity MAP kinase phosphatase | K04459 | DUS1 | 2.0 | 2.9 |
| FLNA | Filamin | K04437 | FLNB | 0.0 | 2.8 |
| MKP | Dual specificity MAP kinase phosphatase | K04459 | DUS2 | 3.6 | 2.6 |
| IL1R | Interleukin 1 receptor | K04386 | IL1R1 | 0.0 | 2.4 |
| HSP72 | Heat shock 70kDa protein 1/2/6/8 | K03283 | HSP71 | 0.9 | 2.4 |
| G12 | Guanine nucleotide-binding protein subunit alpha-12 | K04347 | GBG12 | 0.7 | 2.3 |
| NFκB | Nuclear factor of kappa light polypeptide gene enhancer in B-cells 2 | K04469 | NFKB2 | 0.9 | 2.3 |
| RasGRP | RAS guanyl-releasing protein 1. 2. 3 and 4 | K04350 | GRP4 | 1.7 | 2.3 |
| CACN | Voltage-dependent calcium channel beta-4 | K04865 | CACB4 | 2.9 | 2.2 |
| RAC. CDC42 | RAS-related C3 botulinum toxin substrate 3 | K04393 | RHOU | 0.0 | 2.1 |
| MAPK1/3 | Mitogen-activated protein kinase 1/3 | K04371 | MK15 | -0.1 | 1.9 |
| MAP3K14 | Mitogen-activated protein kinase kinase kinase 14 | K04466 | M3K14 | 1.2 | 1.9 |
| AKT | RAC serine/threonine-protein kinase | K04456 | AKT3 | 0.0 | 1.6 |
| FGF | Fibroblast growth factor | K04358 | FG17 | 0.0 | 1.6 |
| RPS6KA5 | Ribosomal protein S6 kinase alpha-5 | K04445 | KS6A4 | 0.3 | 1.4 |
| MKP | Dual specificity MAP kinase phosphatase | K04459 | DUS6 | 0.0 | 1.4 |
| JUND | Transcription factor jun-D | K04449 | JUND | 2.7 | 1.3 |
| IL1R | Interleukin 1 receptor | K04386 | IL1R2 | 0.0 | 1.3 |
| PAK1&2 | p21-activated kinase 1&2 | K04409 | ZN282 | 0.0 | 1.3 |
| MKP | Dual specificity MAP kinase phosphatase | K04459 | DUS8 | 0.0 | 1.2 |
| CPLA2 | cytosolic phospholipase A2 | K16342 | PA24F | 0.0 | 1.2 |
| PAK1&2 | p21-activated kinase 1&2 | K04409 | ZN423 | 0.0 | 1.2 |
| IKKA | Inhibitor of nuclear factor kappa-B kinase subunit alpha | K04467 | IKKA | 0.0 | 1.2 |
| NR4A1 | Nuclear receptor subfamily 4 group A member 1 | K04465 | NR4A1 | 3.3 | 1.2 |
| JUN | Transcription factor AP-1 | K04448 | JUN | 1.6 | 1.2 |
| FGF | Fibroblast growth factor | K04358 | FGF7 | 0.0 | 1.2 |
| PTPRR | Receptor-type tyrosine-protein phosphatase R | K04458 | PTPRR | 0.0 | 1.2 |
| PTPRR | Dual specificity phosphatase 3 | K18019 | PTN7 | -0.9 | 1.2 |
| GADD45 | Growth arrest and DNA-damage-inducible protein | K04402 | GA45G | 1.6 | 1.1 |
| MAPKAPK2.3&4 | Mitogen-activated protein kinase.activated protein kinase 2 and 3 | K04443 | MAPK2 | 0.4 | 1.1 |
| TAB1 | TAK1-binding protein 1 | K04403 | TAB1 | 0.0 | 1.0 |
| MKP | Dual specificity MAP kinase phosphatase | K04459 | DUS16 | 0.5 | 1.0 |
| CASP | Caspase | K02187 | CASP3 | 0.0 | 1.0 |
| MAP3K2 | Mitogen-activated protein kinase kinase kinase 2 | K04421 | NA | 0.0 | 1.0 |
| IL1R1 | Interleukin 1 receptor | K04386 | IL1RAcP | 0.0 | 0.9 |
| MAP3K13 | Mitogen-activated protein kinase kinase kinase 13 | K04422 | M3K13 | 1.0 | 0.9 |
| TNFSF6 | Tumor necrosis factor ligand superfamily member 6 | K04389 | TNFL6 | 0.0 | 0.9 |
| MNK1/2 | MAP kinase interacting serine/threonine kinase | K04372 | MKNK2 | 1.3 | 0.9 |
| p38 | p38 MAP kinase | K04441 | MK14B | 0.0 | 0.9 |
| PAK1&2 | p21-activated kinase 1&2 | K04409 | ZN226 | 0.0 | 0.9 |
| CACN | Voltage-dependent calcium channel N type alpha - 1C | K04850 | CAC1C | 0.0 | 0.8 |
| MNK1/2 | MAP kinase interacting serine/threonine kinase | K04372 | MOB3C | 0.7 | 0.8 |
| NFκβ | Transcription factor p65 | K04735 | REL | 0.5 | 0.8 |
| RAP1 | RAS-related protein Rap-1A | K04353 | RAP2B | -0.7 | 0.8 |
| STK3&4 | Serine/threonine kinase 3 | K04412 | STK26 | 0.0 | 0.8 |
| RAS | GTPase Kras | K07827 | RASH | 0.0 | 0.8 |
| DAXX | Death-associated protein 6 | K02308 | DAXX | 0.0 | 0.8 |
| NFκβ | Nuclear factor kappa beta | K09253 | RELB | 1.0 | 0.8 |
| RAP1 | RAS-related protein Rap-1B | K07836 | RAP2B | 1.0 | 0.8 |
| ARRB | Beta-arrestin | K04439 | ARR1 | 0.0 | 0.7 |
| STK3&4 | Serine/threonine kinase 3 | K04412 | STK24 | 0.0 | 0.7 |
| MAPKAPK2.3&4 | Mitogen-activated protein kinase.activated protein kinase 4 | K04444 | MAPK3 | 0.0 | 0.7 |
| NFκβ | Transcription factor p65 | K04735 | TF65 | 0.6 | 0.7 |
| GADD45 | Growth arrest and DNA-damage-inducible protein | K04402 | GA45A | 1.3 | 0.7 |
| RAC. CDC42 | Cell division control protein 42 | K04393 | CDC42 | 0.0 | 0.7 |
| MAP3K2 | Mitogen-activated protein kinase kinase kinase 2 | K04421 | CRYD | -1.0 | 0.7 |
| P53 | Tumor protein p53 | K04451 | P53 | 0.4 | 0.7 |
| PPM1B | Protein phosphatase 1B | K04461 | NA | 0.0 | 0.7 |
| ELK-1 | ETS domain-containing protein ELK1 | K04375 | ELK1 | 0.0 | 0.6 |
| AKT | RAC serine/threonine-protein kinase | K04456 | MAST3 | 0.3 | 0.6 |
| NF1 | Neurofibromin | K08052 | NF1 | 0.0 | 0.6 |
| PPP3c | Serine/threonine-protein phosphatase 2B catalytic subunit | K04348 | PP2BB | 0.0 | 0.5 |
| JNK | Mitogen-activated protein kinase 8/9/10 | K04440 | MK09 | 0.0 | 0.5 |
| MAP2K5 | Mitogen-activated protein kinase kinase 5 | K04463 | MP2K5 | -0.7 | 0.5 |
| MAP2K1 | Mitogen-activated protein kinase kinase 1 | K04368 | MP2K1 | 1.4 | 0.5 |
| NFAT4 | Nuclear factor of activated T-cells. cytoplasmic 3 | K17333 | NFAC3 | 0.0 | 0.5 |
| PPP3C | Serine/threonine-protein phosphatase 2B regulatory subunit | K06268 | CHP1 | -0.4 | 0.5 |
| STK3&4 | Serine/threonine kinase 3 | K04411 | STK24 | 0.0 | 0.5 |
| RafB | B-Raf proto-oncogene serine/threonine-protein kinase | K04365 | BRAF | 0.0 | 0.5 |
| NFATC2 | Nuclear factor of activated T-cells. cytoplasmic 1 | K04446 | NFAT5 | 0.0 | 0.5 |
| PP1A | Protein phosphatase 1A | K04457 | PPM1A | 0.0 | 0.4 |
| MAPKAPK5 | Mitogen-activated protein kinase.activated protein kinase 5 | K04442 | MAPK5 | 0.0 | 0.4 |
| NLK | Nemo like kinase | K04468 | NLK2 | 0.0 | 0.4 |
| AKT | RAC serine/threonine-protein kinase | K04456 | AKT2 | 0.0 | 0.4 |
| EVI1 | Ecotropic virus integration site 1 protein | K04462 | ZN236 | 0.0 | 0.3 |
| STK3&4 | Serine/threonine kinase 3 | K04412 | STK3 | 0.0 | 0.3 |
| ELK-4 | ETS domain-containing protein ELK4 | K04376 | ELK3 | 0.0 | 0.3 |
| PPP3c | Serine/threonine-protein phosphatase 2B catalytic subunit | K04348 | PP2BC | 0.0 | 0.3 |
| PAK1&2 | p21-activated kinase 1&2 | K04409 | ZN250 | 0.0 | 0.3 |
| PAK1&2 | p21-activated kinase 1&2 | K04409 | ZN271 | 0.0 | 0.3 |
| JUND | Transcription factor jun-D | K04449 | JUN | 0.6 | 0.2 |
| PAK1&2 | p21-activated kinase 1&2 | K04410 | PAK2 | -0.2 | 0.2 |
| RPS6KA | Ribosomal protein S6 kinase alpha-1/2/3/6 | K04373 | KS6A3 | 0.0 | 0.2 |
| AKT | RAC serine/threonine-protein kinase | K04456 | AKT1 | -0.3 | 0.2 |
| MAP4K3 | Mitogen-activated protein kinase kinase kinase kinase 3 | K04406 | M4K3 | 0.1 | 0.2 |
| TAO | Thousand and one amino acid protein kinase | K04429 | TAOK1 | 0.0 | 0.2 |
| HSP72 | Heat shock 70kDa protein 1/2/6/8 | K03283 | HSP7C | 0.9 | 0.1 |
| p38 | p38 MAP kinase | K04441 | MK14A | -0.3 | 0.1 |
| MAP4K4 | Mitogen-activated protein kinase kinase kinase kinase 4 | K04407 | MINK1 | 0.0 | 0.1 |
| PAK1&2 | p21-activated kinase 1&2 | K04410 | NA | 0.0 | 0.1 |
| PKA | Protein kinase A | K04345 | KAPCA | 0.2 | 0.1 |
| MAX | Max protein | K04453 | MAX | 0.0 | 0.1 |
| IKKA | Inhibitor of nuclear factor kappa-B kinase subunit alpha | K07210 | SPS2 | 0.0 | 0.0 |
| IL1R | Interleukin 1 receptor | K04386 | IL1RAcP | 0.0 | 0.0 |
| PAK1&2 | p21-activated kinase 1&2 | K04409 | PAK1 | 0.0 | 0.0 |
| PAK1&2 | p21-activated kinase 1&2 | K04409 | ZG57 | -0.2 | 0.0 |
| MAPK1/3 | Mitogen-activated protein kinase 1/3 | K04371 | MK01 | 0.0 | 0.0 |
| RPS6KA5 | Ribosomal protein S6 kinase alpha-5 | K04445 | KS6A5 | 0.0 | 0.0 |
| MAPK7 | Mitogen-activated protein kinase 7 | K04464 | MK07 | 0.0 | 0.0 |
| TAO | Thousand and one amino acid protein kinase | K04429 | TAOK2 | 0.8 | 0.0 |
| ARRB | Beta-arrestin | K04439 | ARRB1 | 0.1 | 0.0 |
| CACN | Voltage-dependent calcium channel N type alpha - 1H | K04855 | CAC1H | 0.0 | 0.0 |
| CACN | Voltage-dependent calcium channel T type alpha - 1I | K04856 | PPR29 | 0.0 | 0.0 |
| CACN | Voltage-dependent calcium channel L tyoe alpha - 1S | K04857 | CAC1S | 0.0 | 0.0 |
| CACN | Voltage-dependent calcium channel alpha-2/delta-2 | K04859 | CA2D2 | 0.0 | 0.0 |
| CACN | Voltage-dependent calcium channel alpha-2/delta-4 | K04861 | NA | 0.0 | 0.0 |
| CACN | Voltage-dependent calcium channel gamma-7 | K04872 | CCG7 | 0.0 | 0.0 |
| CPLA2 | cytosolic phospholipase A2 | K16342 | JMJD7 | 0.0 | 0.0 |
| CREB | Cyclic AMP-dependent transcription factor ATF-4 | K04374 | ATF4 | 0.1 | 0.0 |
| CREBP1 | Cyclic AMP-dependent transcription factor ATF-2 | K04450 | ATF2 | 0.0 | 0.0 |
| CREBP1 | Cyclic AMP-dependent transcription factor ATF-2 | K04450 | ATF7 | 0.0 | 0.0 |
| CRK II | Proto-oncogene C-crk | K04438 | CRKL | 0.0 | 0.0 |
| ELK-4 | ETS domain-containing protein ELK4 | K04376 | ELK4 | 0.0 | 0.0 |
| ELK-4 | ETS domain-containing protein ELK4 | K04376 | FURIN | 0.0 | 0.0 |
| FGF | Fibroblast growth factor | K04358 | FGF19 | 0.0 | 0.0 |
| FGF | Fibroblast gowth factor 1 | K18496 | NFI1L | 0.0 | 0.0 |
| FGFR1 | Fibroblast growth factor receptor 2 | K05093 | PTK7 | 0.0 | 0.0 |
| FOS | Proto-oncogene protein c-fos | K04379 | FOS | 2.6 | 0.0 |
| GRB2 | Growth factor receptor-binding protein 2 | K04364 | GRB2 | -0.4 | 0.0 |
| HSP72 | Heat shock 70kDa protein 1/2/6/8 | K03283 | GIN1 | 0.0 | 0.0 |
| HSP72 | Heat shock 70kDa protein 1/2/6/8 | K03283 | HSP70 | 0.0 | 0.0 |
| IKKA | Inhibitor of nuclear factor kappa-B kinase subunit alpha | K07210 | OPTN | -0.4 | 0.0 |
| IL1R1 | Interleukin 1 receptor | K04386 | IL1R1 | 0.0 | 0.0 |
| JIP3 | Mitogen-activated protein kinase 8 interacting protein 3 | K04436 | JIP3 | 0.0 | 0.0 |
| MAP2K2 | Mitogen-activated protein kinase kinase 2 | K04369 | MP2K2 | -0.4 | 0.0 |
| MAP2K7 | Mitogen-activated protein kinase kinase 4 | K04431 | MP2K7 | -0.3 | 0.0 |
| MAP3K11 | Mitogen-activated protein kinase kinase kinase 11 | K04419 | M3K11 | 0.0 | 0.0 |
| MAP3K11 | Mitogen-activated protein kinase kinase kinase 11 | K04419 | M3KL4 | 0.0 | 0.0 |
| MAP3K2 | Mitogen-activated protein kinase kinase kinase 2 | K04420 | M3K19 | 0.0 | 0.0 |
| MAP3K7 | Mitogen-activated protein kinase kinase kinase 7 | K04427 | M3K7 | -0.3 | 0.0 |
| MAP3K8 | Mitogen-activated protein kinase kinase kinase 8 | K04415 | M3K8 | 0.7 | 0.0 |
| MAP4K1 | Mitogen-activated protein kinase kinase kinase kinase 1 | K04408 | M4K5 | 0.0 | 0.0 |
| MAP4K2 | Mitogen-activated protein kinase kinase kinase kinase 2 | K04414 | M4K5 | 0.0 | 0.0 |
| MAP4K2 | Mitogen-activated protein kinase kinase kinase kinase 2 | K04414 | RBM41 | -0.5 | 0.0 |
| MAP4K3 | Mitogen-activated protein kinase kinase kinase kinase 3 | K04406 | M4K5 | -0.3 | 0.0 |
| MAPK1/3 | Mitogen-activated protein kinase 1/3 | K04371 | MK03 | -0.6 | 0.0 |
| MAPK1/3 | Mitogen-activated protein kinase 1/3 | K04371 | MK06 | 0.0 | 0.0 |
| MAX | Max protein | K04453 | NA | 0.0 | 0.0 |
| MKP | Dual specificity MAP kinase phosphatase | K04459 | DUS5 | 0.9 | 0.0 |
| MKP | Dual specificity protein phosphatases | K20216 | S17A5 | 0.8 | 0.0 |
| MOS | Proto-oncogene serine/threonine-protein kinase mos | K04367 | MOS | 0.0 | 0.0 |
| MYC | Myc proto-oncogene protein | K04377 | MYC2 | 0.0 | 0.0 |
| NFκβ | Nuclear factor of kappa light polypeptide gene enhancer in B-cells 2 | K04469 | DHE3 | 0.6 | 0.0 |
| NFκβ | Nuclear factor kappa beta | K02580 | NFKB1 | 0.0 | 0.0 |
| p120GAF | RAS GTPase-activating protein 1 | K04352 | RASA1 | -0.6 | 0.0 |
| p38 | p38 MAP kinase | K04441 | MK11 | 0.3 | 0.0 |
| p38 | p38 MAP kinase | K04441 | SELO | 0.0 | 0.0 |
| PAK1&2 | p21-activated kinase 1&2 | K04409 | ZN208 | 0.0 | 0.0 |
| PAK1&2 | p21-activated kinase 1&2 | K04409 | ZN234 | 0.0 | 0.0 |
| PAK1&2 | p21-activated kinase 1&2 | K04409 | ZN521 | 0.0 | 0.0 |
| PAK1&2 | p21-activated kinase 1&2 | K04409 | ZN574 | 0.0 | 0.0 |
| PAK1&2 | p21-activated kinase 1&2 | K04409 | ZN793 | 0.0 | 0.0 |
| PKA | Protein kinase A | K04345 | PRKX | 0.0 | 0.0 |
| PPM1B | Protein phosphatase 1B | K04461 | PPM1B | 0.0 | 0.0 |
| PPP5C | Serine/threonine-protein phosphatase 5 | K04460 | PPP5 | -0.5 | 0.0 |
| RPS6KA | Ribosomal protein S6 kinase alpha-1/2/3/6 | K04373 | KS6A6 | 0.4 | 0.0 |
| RPS6KA | Ribosomal protein S6 kinase alpha-1/2/3/6 | K04373 | KS6AA | -0.5 | 0.0 |
| SOS | Son of sevenless | K03099 | RGPS1 | 0.0 | 0.0 |
| SOS | Son of sevenless | K03099 | SOS1 | 0.0 | 0.0 |
| STK3&4 | Serine/threonine kinase 3 | K04411 | OXSR1 | 0.0 | 0.0 |
| STK3&4 | Serine/threonine kinase 3 | K04411 | STK4 | 0.0 | 0.0 |
| STK3&4 | Serine/threonine kinase 3 | K04412 | OXSR1 | -1.7 | 0.0 |
| STK3&4 | Serine/threonine kinase 3 | K04412 | STK25 | 0.0 | 0.0 |
| TAB2 | TAK1-binding protein 2 | K04404 | TAB2 | 0.0 | 0.0 |
| TAO | Thousand and one amino acid protein kinase | K04429 | NA | 0.0 | 0.0 |
| TGFBR | TGF-beta receptor type-2 | K04674 | TGFR1 | 0.0 | 0.0 |
| TNF | Tumor necrosis factor | K03156 | TNFB | 0.0 | 0.0 |
| PPP3C | Serine/threonine-protein phosphatase 2B catalytic subunit | K04348 | PP2BA | 0.0 | 0.0 |
| TGFBR | TGF-beta receptor type-2 | K04388 | TGFR2 | 0.0 | 0.0 |
| PAK1&2 | p21-activated kinase 1&2 | K04409 | ZN273 | 0.0 | 0.0 |
| TRAF6 | TNF receptor-associated factor 6 | K03175 | TRAF6 | 0.0 | 0.0 |
| CRK II | Proto-oncogene C-crk | K04438 | CRK | 0.0 | 0.0 |
| PAK1&2 | p21-activated kinase 1&2 | K04409 | ZN665 | 0.0 | 0.0 |
| ESCIT | Evolutionarily conserved signaling indermediate in Toll pathway | K04405 | ECSIT | 0.0 | 0.0 |
| HSP72 | Heat shock 70kDa protein 1/2/6/8 | K03283 | HSP7E | 0.4 | 0.0 |
| RAC. CDC42 | RAS-related C3 botulinum toxin substrate 1 | K04392 | RAC1 | 0.0 | 0.0 |
| PPP3C | Serine/threonine-protein phosphatase 2B regulatory subunit | K06268 | CANB1 | 0.3 | 0.0 |
| PAK1&2 | p21-activated kinase 1&2 | K04409 | ZN433 | 0.0 | 0.0 |
| MYC | Myc proto-oncogene protein | K04377 | MYC | 0.0 | 0.0 |
| PAK1&2 | p21-activated kinase 1&2 | K04409 | Z658B | 0.0 | -0.1 |
| PAK1&2 | p21-activated kinase 1&2 | K04409 | ZN160 | 0.0 | -0.1 |
| PAK1&2 | p21-activated kinase 1&2 | K04409 | ZN835 | -0.9 | -0.1 |
| PAK1&2 | p21-activated kinase 1&2 | K04409 | ZSCA2 | 0.0 | -0.1 |
| AKT | RAC serine/threonine-protein kinase | K04456 | AKT2A | 0.0 | -0.1 |
| JIP3 | Mitogen-activated protein kinase 8 interacting protein 3 | K04436 | JIP4 | 0.0 | -0.1 |
| MAP4K4 | Mitogen-activated protein kinase kinase kinase kinase 4 | K04407 | M4K4 | -0.1 | -0.1 |
| PAK1&2 | p21-activated kinase 1&2 | K04409 | ZN724 | -0.1 | -0.1 |
| PKC | Classical protein kinase C | K02677 | KPCA | -0.2 | -0.2 |
| MAP4K2 | Mitogen-activated protein kinase kinase kinase kinase 2 | K04414 | RBM4 | 0.0 | -0.2 |
| CPLA2 | cytosolic phospholipase A2 | K16342 | PA24A | -1.2 | -0.2 |
| MAP4K4 | Mitogen-activated protein kinase kinase kinase kinase 4 | K04407 | TNIK | -0.2 | -0.3 |
| PAK1&2 | p21-activated kinase 1&2 | K04409 | ZN235 | 0.0 | -0.3 |
| RAC. CDC42 | RAS-related C3 botulinum toxin substrate 2 | K04392 | RAC2 | 0.3 | -0.3 |
| MAP2K4 | Mitogen-activated protein kinase kinase 4 | K04430 | MP2K4 | 0.0 | -0.3 |
| RAC. CDC42 | Ras-related C3 botulinum toxin substrate 1 | K07861 | RAC1 | 0.0 | -0.4 |
| TGFB | Transforming growth factor beta-1 | K13375 | TGFB1 | 0.7 | -0.4 |
| RAPGEF2 | Rap guanine nucleotide exchange factor 2 | K08018 | RPGF2 | 0.3 | -0.4 |
| MAP4K2 | Mitogen-activated protein kinase kinase kinase kinase 2 | K04414 | RBM4B | 0.0 | -0.5 |
| MAP4K4 | Mitogen-activated protein kinase kinase kinase kinase 4 | K04407 | NA | 1.0 | -0.5 |
| PAK1&2 | p21-activated kinase 1&2 | K04409 | ZN652 | -0.1 | -0.5 |
| PAK1&2 | p21-activated kinase 1&2 | K04409 | ZNF91 | 0.0 | -0.5 |
| PKA | Protein kinase A | K04345 | KAPCB | 0.0 | -0.5 |
| PPM1B | Protein phosphatase 1B | K04461 | PPM1G | 0.0 | -0.5 |
| PDGFR | Platelet-derived growth factor receptor beta | K05089 | PGFRB | -0.6 | -0.5 |
| NFATC2 | Nuclear factor of activated T-cells. cytoplasmic 1 | K04446 | NFAC1 | 0.0 | -0.5 |
| RPS6KA | Ribosomal protein S6 kinase alpha-1/2/3/6 | K04373 | ST32C | 0.0 | -0.5 |
| GADD45 | Growth arrest and DNA-damage-inducible protein | K04402 | GA45B | -0.4 | -0.5 |
| NFκβ | Nuclear factor of K light polypeptide gene enhancer in B-cells 2 | K04469 | FA35A | 0.4 | -0.6 |
| AKT | RAC serine/threonine-protein kinase | K04456 | NA | 0.0 | -0.6 |
| p38 | p38 MAP kinase | K04441 | MK13 | -0.6 | -0.6 |
| MKP | Dual specificity MAP kinase phosphatase | K04459 | DUS7 | -1.0 | -0.6 |
| PKC | Classical protein kinase C beta type | K19662 | KPCB | 0.0 | -0.7 |
| JNK | Mitogen-activated protein kinase 8/9/10 | K04440 | MK08 | 0.0 | -0.7 |
| RasGRP | RAS guanyl-releasing protein 1. 2. 3 and 4 | K04350 | GRP1 | 0.0 | -0.7 |
| PAK1&2 | p21-activated kinase 1&2 | K04409 | ZBT11 | 0.0 | -0.8 |
| ZAK | Sterile alpha motif and leucine zipper containing kinase AZK | K04424 | MLTK | 0.0 | -0.8 |
| CACN | Voltage-dependent calcium channel N type alpha - 1D | K04851 | CAC1D | 0.0 | -0.8 |
| PAK1&2 | p21-activated kinase 1&2 | K04409 | ZBT24 | 0.0 | -0.8 |
| CDC25B | M-phase inducer phosphatase 2 | K05866 | MPIP2 | 0.0 | -0.8 |
| TAO | Thousand and one amino acid protein kinase | K04429 | TAOK3 | 0.0 | -0.8 |
| PAK1&2 | p21-activated kinase 1&2 | K04409 | XFIN | 0.0 | -0.9 |
| RPS6KA5 | Ribosomal protein S6 kinase alpha-5 | K04445 | SG494 | 0.0 | -0.9 |
| STMN1 | Stathmin | K04381 | STMN1 | 0.0 | -0.9 |
| RASGRF | Ras-specific guanine nucleotide-releasing factor 2 | K12326 | RGRF2 | 0.2 | -0.9 |
| RAS | RAS-related protein R-Ras2 | K07830 | RRAS | 0.0 | -1.0 |
| MAP3K4 | Mitogen-activated protein kinase kinase kinase 4 | K04428 | M3K4 | 0.0 | -1.0 |
| MKP | Dual specificity MAP kinase phosphatase | K04459 | DUS4 | 0.0 | -1.0 |
| CACN | Voltage-dependent calcium channel alpha-2/delta-4 | K04861 | CA2D4 | -1.6 | -1.0 |
| TRAF2 | TNF receptor-associated factor 2 | K03173 | TRAF2 | -0.8 | -1.1 |
| MAP3K2 | Mitogen-activated protein kinase kinase kinase 2 | K04420 | M3K2 | -0.5 | -1.1 |
| MAP3K2 | Mitogen-activated protein kinase kinase kinase 2 | K04421 | M3K3 | -0.5 | -1.1 |
| TNFR | Tumor necrosis factor receptor | K03158 | TNR1A | -2.5 | -1.1 |
| SRF | Serum response factor | K04378 | SRF | 1.1 | -1.2 |
| MEF2C | MADS-box transcription enhancer factor 2C | K04454 | MEF2C | -0.5 | -1.2 |
| FGFR1 | Fibroblast growth factor receptor 2 | K05094 | FGRL1 | -2.4 | -1.2 |
| TNFR | Tumor necrosis factor receptor | K03158 | NA | -0.8 | -1.3 |
| CACN | Voltage-dependent calcium channel alpha-2/delta-3 | K04860 | CA2D3 | 0.0 | -1.4 |
| TNFSF6 | Tumor necrosis factor ligand superfamily member 6 | K04389 | NA | 0.0 | -1.5 |
| FLNA | Filamin | K04437 | FLNC | 0.0 | -1.6 |
| p38 | p38 MAP kinase | K04441 | MK12 | 0.0 | -1.6 |
| RPS6KA5 | Ribosomal protein S6 kinase alpha-5 | K04445 | ST32A | 0.0 | -1.7 |
| PTPRR | Dual specificity phosphatase 3 | K17614 | DUS27 | 0.0 | -1.7 |
| RASGRP | RAS guanyl-releasing protein 2 | K12361 | GRP2 | -0.6 | -1.8 |
| MAP3K5 | Mitogen-activated protein kinase kinase kinase 5 | K04426 | M3K5 | -0.1 | -1.8 |
| MAP2K6 | Mitogen-activated protein kinase kinase 6 | K04433 | MP2K6 | 0.0 | -1.9 |
| NTRK | Neurotrophic tyrosine kinase receptor | K04360 | DDR2 | -1.0 | -2.0 |
| JIP1&2 | Mitogen-activated protein kinase 8 interacting protein 1 | K04434 | JIP1 | -1.8 | -2.2 |
| FLNA | Filamin | K04437 | FL. | 0.0 | -2.2 |
| CACN | Voltage-dependent calcium channel N type alpha - 1B | K04849 | CAC1B | 0.0 | -2.2 |
| NFκβ | Transcription factor p65 | K04735 | NA | 0.0 | -2.2 |
| GRB2 | Growth factor receptor-binding protein 2 | K04364 | GRAP | 0.0 | -2.5 |
| RAP1 | RAS-related protein Rap-1A | K04353 | RAP1B | 0.0 | -2.5 |
| RAS | RAS-related protein R-Ras2 | K07830 | RRAS2 | 0.0 | -2.6 |
| RASA2 | RAS GTPase-activating protein 2 | K08053 | RASA2 | 0.0 | -2.6 |
| PDGF | Platelet-derived growth factor subunit B | K04359 | PDGFB | -5.0 | -3.0 |
| MAP3K12 | Mitogen-activated protein kinase kinase kinase 12 | K04423 | M3K12 | 0.0 | -3.9 |
| TGFB | Transforming growth factor beta-3 | K13377 | TGFB3 | -3.2 | -4.1 |
| MAPK1/3 | Mitogen-activated protein kinase 1/3 | K04371 | MK04 | -1.9 | -5.2 |
