# Supplementary material for: Interleukin-1 Ligands and Receptors in Lumpfish (Cyclopterus lumpus L.): Molecular Characterization, Phylogeny, Gene Expression, and Transcriptome Analyses
Source: Front Immunol. 2020 Apr 2;11:502. doi: 10.3389/fimmu.2020.00502 (PMC7144542; doi:10.3389/fimmu.2020.00502)
Supplement: Supplemental Table 4 — Sequences included in the phylogenetic tree. [file Table_4.docx]

| **Suplemental Table 4**. Sequences included in the phylogenetic tree | | |
| --- | --- | --- |
| **GI number** | **Full name** | **Specie** |
| **IL-18** |  |  |
| gi\|1007733581 | PREDICTED: uncharacterized protein LOC107380752 | *Nothobranchius furzeri* |
| gi\|1039366873 | PREDICTED: interleukin-18 isoform X1 | *Poecilia reticulata* |
| gi\|1041133957 | interleukin-18 isoform X1 | *Kryptolebias marmoratus* |
| gi\|1041133959 | interleukin-18 isoform X2 | *Kryptolebias marmoratus* |
| gi\|1108913370 | PREDICTED: uncharacterized protein LOC104940571 | *Larimichthys crocea* |
| gi\|1110897243 | uncharacterized protein LOC109205098 isoform X3 | *Oreochromis niloticus* |
| gi\|1110897245 | uncharacterized protein LOC109205098 isoform X4 | *Oreochromis niloticus* |
| gi\|1130004025 | PREDICTED: uncharacterized protein LOC109510913 | *Hippocampus comes* |
| gi\|1148294668 | IL-18 | *Miichthys miiuy* |
| gi\|1158907633 | uncharacterized protein LOC109905603 | *Oncorhynchus kisutch* |
| gi\|1168915311 | uncharacterized protein LOC109992315 isoform X1 | *Labrus bergylta* |
| gi\|1168915313 | uncharacterized protein LOC109992315 isoform X2 | *Labrus bergylta* |
| gi\|1211326050 | interleukin 18 isoform X1 | *Oncorhynchus mykiss* |
| gi\|1211326052 | interleukin 18 isoform X2 | *Oncorhynchus mykiss* |
| gi\|1229002985 | interleukin-18-like | *Acanthochromis polyacanthus* |
| gi\|1316063835 | interleukin-18 isoform X1 | *Xiphophorus maculatus* |
| gi\|1334664277 | interleukin-18 | *Trichechus manatus latirostris* |
| gi\|1343040406 | uncharacterized protein LOC111949364 | *Salvelinus alpinus* |
| gi\|1389919994 | uncharacterized protein LOC112486949 isoform X1 | *Cynoglossus semilaevis* |
| gi\|1389919996 | uncharacterized protein LOC112486949 isoform X2 | *Cynoglossus semilaevis* |
| gi\|1395226856 | Hypothetical protein SMAX5B 8461 | *Scophthalmus maximus* |
| gi\|1395226857 | Hypothetical protein SMAX5B 8461 | *Scophthalmus maximus* |
| gi\|1434964500 | uncharacterized protein LOC109205098 isoform X1 | *Oreochromis niloticus* |
| gi\|1434964502 | uncharacterized protein LOC109205098 isoform X2 | *Oreochromis niloticus* |
| gi\|185133182 | interleukin 18 | *Oncorhynchus mykiss* |
| gi\|209736822 | Interleukin-18 precursor | *Salmo salar* |
| gi\|213515160 | Interleukin-18 | *Salmo salar* |
| gi\|221220822 | Interleukin-18 precursor | *Salmo salar* |
| gi\|303664145 | Interleukin-18 precursor | *Salmo salar* |
| gi\|45260639 | interleukin-18 | *Takifugu rubripes* |
| gi\|45260642 | interleukin-18 | *Takifugu rubripes* |
| gi\|50080007 | interleukin 18 form a, IL-18A | *Oncorhynchus mykiss* |
| gi\|50080008 | interleukin 18 form b, IL-18B | *Oncorhynchus mykiss* |
| gi\|529125499 | interleukin-18 | *Sparus aurata* |
| gi\|551511472 | interleukin-18 isoform X2 | *Xiphophorus maculatus* |
| gi\|617495115 | PREDICTED: interleukin-18-like | *Poecilia formosa* |
| gi\|657547446 | PREDICTED: interleukin-18-like | *Stegastes partitus* |
| gi\|658881169 | PREDICTED: interleukin-18 isoform X2 | *Poecilia reticulata* |
| gi\|74095937 | interleukin-18 | *Takifugu rubripes* |
| gi\|742249475 | PREDICTED: interleukin-18 | *Esox lucius* |
| gi\|958906940 | IL18 | *Poeciliopsis prolifica* |
| gi\|961974860 | PREDICTED: interleukin-18 | *Poecilia latipinna* |
| gi\|962020624 | PREDICTED: interleukin-18-like | *Poecilia mexicana* |
| gi\|974117749 | PREDICTED: interleukin-18-like | *Cyprinodon variegatus* |
| P70380 | IL18 MOUSE Interleukin-18 | *Mus musculus* |
| Q14116 | IL18 HUMAN Interleukin-18 | *Homo sapiens* |
| TBA | IL18 | *Cyclopterus lumpus* |
| **IL-1b** |  |  |
| gi\|1049222026 | PREDICTED: interleukin-1 beta-like | *Pygocentrus nattereri* |
| gi\|1049222285 | PREDICTED: interleukin-1 beta-like | *Pygocentrus nattereri* |
| gi\|1049222287 | PREDICTED: interleukin-1 beta-like | *Pygocentrus nattereri* |
| gi\|1083270956 | PREDICTED: interleukin-1 beta | *Scleropages formosus* |
| gi\|1158862069 | interleukin-1 beta | *Oncorhynchus kisutch* |
| gi\|1158883640 | interleukin-1 beta-like | *Oncorhynchus kisutch* |
| gi\|1158946287 | interleukin-1 beta-like | *Oncorhynchus kisutch* |
| gi\|12049717 | interleukin 1 beta 43467 | *Cyprinus carpio* |
| gi\|12049719 | interleukin 1 beta 43498 | *Cyprinus carpio* |
| gi\|1211247410 | interleukin-1 beta-like | *Oncorhynchus mykiss* |
| gi\|1211332815 | interleukin-1 beta-like | *Oncorhynchus mykiss* |
| gi\|1211353172 | interleukin-1 beta-like | *Oncorhynchus mykiss* |
| gi\|1237948414 | interleukin 1 beta | *Gymnocypris eckloni* |
| gi\|1249021769 | interleukin-1 beta-like | *Astyanax mexicanus* |
| gi\|1249021771 | interleukin-1 beta-like | *Astyanax mexicanus* |
| gi\|1338771276 | interleukin-1 beta-like | *Paramormyrops kingsleyae* |
| gi\|1342968161 | interleukin-1 beta | *Salvelinus alpinus* |
| gi\|1343017407 | interleukin-1 beta | *Salvelinus alpinus* |
| gi\|1348556044 | interleukin-1 beta-like | *Salvelinus alpinus* |
| gi\|1367437602 | interleukin-1 beta | *Oncorhynchus tshawytscha* |
| gi\|1367506101 | interleukin-1 beta-like isoform X1 | *Oncorhynchus tshawytscha* |
| gi\|1367506105 | interleukin-1 beta-like isoform X2 | *Oncorhynchus tshawytscha* |
| gi\|1367536039 | interleukin-1 beta-like | *Oncorhynchus tshawytscha* |
| gi\|144225831 | interleukin-1 beta | *Melanogrammus aeglefinus* |
| gi\|1450278189 | interleukin 1 beta | *Tachysurus fulvidraco* |
| gi\|1503276292 | interleukin-1 beta-like | *Pangasianodon hypophthalmus* |
| gi\|152962704 | interleukin-1beta | *Conger myriaster* |
| gi\|157652606 | interleukin 1b | *Gadus morhua* |
| gi\|164510038 | interleukin-1 beta | *Salvelinus alpinus* |
| gi\|165929363 | interleukin 1b | *Melanogrammus aeglefinus* |
| gi\|18152761 | interleukin-1 beta | *Oncorhynchus mykiss* |
| gi\|185133434 | interleukin-1 beta | *Oncorhynchus mykiss* |
| gi\|186288128 | interleukin-1 beta | *Salmo salar* |
| gi\|25137090 | interleukin-1 beta-1 | *Carassius auratus* |
| gi\|25137092 | interleukin-1 beta-2 | *Carassius auratus* |
| gi\|2821975 | interleukin-1 beta | *Cyprinus carpio* |
| gi\|307075895 | interlukin-1 beta | *Danio rerio* |
| gi\|317414915 | interleukin 1 beta | *Danio rerio* |
| gi\|317574215 | interleukin 1 beta | *Ictalurus punctatus* |
| gi\|318098733 | interleukin-1 beta | *Ictalurus punctatus* |
| gi\|33356628 | interleukin 1 beta | *Danio rerio* |
| gi\|3805826 | interleukin-1 beta | *Oncorhynchus mykiss* |
| gi\|3805831 | interleukin-1-beta | *Oncorhynchus mykiss* |
| gi\|38143017 | interleukin-1 beta 1 | *Carassius auratus* |
| gi\|38143019 | interleukin-1 beta 2 | *Carassius auratus* |
| gi\|387864279 | interleukin-1 beta | *Ctenopharyngodon idella* |
| gi\|387864328 | interleukin-1 beta | *Ctenopharyngodon idella* |
| gi\|390483256 | IL-1 beta | *Hemibagrus macropterus* |
| gi\|393010847 | interleukin-1 beta | *Ctenopharyngodon idella* |
| gi\|431831911 | interleukin-1 beta | *Cyprinus carpio* |
| gi\|47607481 | interleukin-1 beta | *Salmo salar* |
| gi\|487395370 | Interleukin-1 beta | *Plecoglossus altivelis* |
| gi\|498917176 | interleukin-1 beta | *Danio rerio* |
| gi\|507104794 | interleukin-1 b3 | *Salmo salar* |
| gi\|536720426 | interleukin-1 beta-1 | *Carassius carassius* |
| gi\|536720431 | interleukin-1 beta-2 | *Carassius carassius* |
| gi\|5708097 | interleukin-1-beta | *Cyprinus carpio* |
| gi\|571255055 | interleukin-1 beta | *Ictalurus punctatus* |
| gi\|571255097 | interleukin-1 beta | *Gadus morhua* |
| gi\|571255099 | Interleukin-1 beta 3 | *Oncorhynchus mykiss* |
| gi\|571255109 | Interleukin-1 beta 3 | *Oncorhynchus mykiss* |
| gi\|57283085 | interleukin 1 beta | *Gadus morhua* |
| gi\|576887285 | Interleukin-1 beta | *Ictalurus punctatus* |
| gi\|642003711 | unnamed protein product | *Oncorhynchus mykiss* |
| gi\|642055264 | unnamed protein product | *Oncorhynchus mykiss* |
| gi\|642085950 | unnamed protein product | *Oncorhynchus mykiss* |
| gi\|642112014 | unnamed protein product | *Oncorhynchus mykiss* |
| gi\|6468654 | interleukin-1 beta 2 precursor | *Oncorhynchus mykiss* |
| gi\|68534031 | Il1b protein | *Danio rerio* |
| gi\|698320871 | interleukin 1 beta | *Coregonus maraena* |
| gi\|74027236 | interleukin 1 beta 1 | *Ictalurus punctatus* |
| gi\|74027238 | interleukin 1 beta 2 | *Ictalurus punctatus* |
| gi\|742245874 | PREDICTED: interleukin-1 beta | *Esox lucius* |
| gi\|742245876 | PREDICTED: interleukin-1 beta | *Esox lucius* |
| gi\|742250859 | PREDICTED: interleukin-1 beta isoform X1 | *Esox lucius* |
| gi\|742250861 | PREDICTED: interleukin-1 beta isoform X2 | *Esox lucius* |
| gi\|78707325 | interleukin 1 beta type a | *Ictalurus punctatus* |
| gi\|78707327 | interleukin 1 beta type b | *Ictalurus punctatus* |
| gi\|78707329 | interleukin 1 beta type a | *Ictalurus punctatus* |
| gi\|78707331 | interleukin 1 beta type b | *Ictalurus punctatus* |
| gi\|8249932 | interleukin-1 beta | *Oncorhynchus mykiss* |
| gi\|831271282 | PREDICTED: interleukin-1 beta-like | *Clupea harengus* |
| gi\|831271284 | PREDICTED: interleukin-1 beta-like | *Clupea harengus* |
| gi\|831271286 | PREDICTED: interleukin-1 beta-like | *Clupea harengus* |
| gi\|929156400 | PREDICTED: interleukin-1 beta-like | *Salmo salar* |
| gi\|929245524 | PREDICTED: interleukin-1 beta-like | *Salmo salar* |
| gi\|929297216 | PREDICTED: interleukin-1 beta isoform X1 | *Salmo salar* |
| gi\|938051386 | interleukin-1 beta-like | *Scleropages formosus* |
| P01584 | IL1B HUMAN Interleukin-1 beta | *Homo sapiens* |
| P10749 | IL1B MOUSE Interleukin-1 beta | *Mus musculus* |
| TBA | Interleukin 1 beta | *Cyclopterus lumpus* |
| **IL-1Fm2** |  |  |
| gi\|1025472254 | PREDICTED: interleukin-1 beta-like isoform X1 | *Poecilia formosa* |
| gi\|1025472256 | PREDICTED: interleukin-1 beta-like isoform X2 | *Poecilia formosa* |
| gi\|1079714444 | PREDICTED: interleukin-1 beta-like | *Lates calcarifer* |
| gi\|1143368044 | PREDICTED: interleukin-1 beta-like | *Paralichthys olivaceus* |
| gi\|1168937023 | interleukin-1 beta-like isoform X1 | *Labrus bergylta* |
| gi\|1168937025 | interleukin-1 beta-like isoform X2 | *Labrus bergylta* |
| gi\|1169063128 | interleukin-1 beta-like | *Monopterus albus* |
| gi\|1188083442 | interleukin-1 beta-like | *Boleophthalmus pectinirostris* |
| gi\|1199311892 | interleukin-1 beta isoform X2 | *Fundulus heteroclitus* |
| gi\|1228982676 | interleukin-1 beta-like | *Acanthochromis polyacanthus* |
| gi\|1250169094 | interleukin-1 beta-like | *Seriola dumerili* |
| gi\|1308535136 | interleukin-1 beta-like isoform X1 | *Amphiprion ocellaris* |
| gi\|1308535175 | interleukin-1 beta-like isoform X2 | *Amphiprion ocellaris* |
| gi\|1316107519 | interleukin-1 beta isoform X2 | *Xiphophorus maculatus* |
| gi\|1317113170 | interleukin-1 beta-like | *Seriola lalandi dorsalis* |
| gi\|1343910006 | interleukin-1 beta | *Oryzias latipes* |
| gi\|1357736833 | interleukin-1 beta-like isoform X1 | *Oryzias melastigma* |
| gi\|1357736835 | interleukin-1 beta-like isoform X2 | *Oryzias melastigma* |
| gi\|1357736837 | interleukin-1 beta-like isoform X3 | *Oryzias melastigma* |
| gi\|1381445067 | interleukin-1 beta isoform X1 | *Maylandia zebra* |
| gi\|1387734041 | hypothetical protein CCH79 1250 | *Gambusia affinis* |
| gi\|1395229004 | Interleukin 1 beta-like 1 | *Scophthalmus maximus* |
| gi\|1470014534 | interleukin-1 beta-like isoform X1 | *Mastacembelus armatus* |
| gi\|1472967553 | interleukin-1 beta-like | *Anabas testudineus* |
| gi\|225706200 | Interleukin-1 beta precursor | *Osmerus mordax* |
| gi\|422001748 | interleukin 1 beta-like 1 | *Paralichthys olivaceus* |
| gi\|422001750 | interleukin 1 beta-like 1 | *Paralichthys olivaceus* |
| gi\|542241173 | interleukin-1 beta | *Oreochromis niloticus* |
| gi\|571257110 | interleukin-1 family member 2 | *Sparus aurata* |
| gi\|584004675 | PREDICTED: interleukin-1 beta-like isoform X1 | *Neolamprologus brichardi* |
| gi\|584004677 | PREDICTED: interleukin-1 beta-like isoform X2 | *Neolamprologus brichardi* |
| gi\|657588828 | PREDICTED: interleukin-1 beta-like | *Stegastes partitus* |
| gi\|657793760 | interleukin-1 beta-like | *Cynoglossus semilaevis* |
| gi\|658876923 | PREDICTED: interleukin-1 beta-like isoform X1 | *Poecilia reticulata* |
| gi\|658876925 | PREDICTED: interleukin-1 beta-like isoform X2 | *Poecilia reticulata* |
| gi\|736195106 | PREDICTED: interleukin-1 beta-like | *Notothenia coriiceps* |
| gi\|808860934 | Interleukin-1 beta | *Larimichthys crocea* |
| gi\|831485658 | interleukin-1 beta isoform X1 | *Fundulus heteroclitus* |
| gi\|928043369 | PREDICTED: interleukin-1 beta-like | *Austrofundulus limnaeus* |
| gi\|930773371 | PREDICTED: interleukin-1 beta-like | *Haplochromis burtoni* |
| gi\|941812403 | interleukin-1 beta isoform X1 | *Xiphophorus maculatus* |
| gi\|961793134 | PREDICTED: interleukin-1 beta-like isoform X1 | *Poecilia mexicana* |
| gi\|961793136 | PREDICTED: interleukin-1 beta-like isoform X2 | *Poecilia mexicana* |
| gi\|961847762 | PREDICTED: interleukin-1 beta-like isoform X1 | *Poecilia latipinna* |
| gi\|961847766 | PREDICTED: interleukin-1 beta-like isoform X2 | *Poecilia latipinna* |
| gi\|974107253 | PREDICTED: interleukin-1 beta-like | *Cyprinodon variegatus* |
| TBA | Interleukin 1 family member 2 | *Cyclopterus lumpus* |
| **nIL-1F1** |  |  |
| gi\|1007736865 | PREDICTED: uncharacterized protein LOC107381689 | *Nothobranchius furzeri* |
| gi\|1020401635 | PREDICTED: uncharacterized protein LOC107555915 | *Sinocyclocheilus grahami* |
| gi\|1025123909 | PREDICTED: uncharacterized protein LOC107695052 | *Sinocyclocheilus anshuiensis* |
| gi\|1025185898 | PREDICTED: uncharacterized protein LOC107751154 | *Sinocyclocheilus rhinocerous* |
| gi\|1025387253 | PREDICTED: uncharacterized protein LOC107737257 | *Sinocyclocheilus rhinocerous* |
| gi\|1039391223 | PREDICTED: uncharacterized protein LOC103462027 isoform X1 | *Poecilia reticulata* |
| gi\|1039391225 | PREDICTED: uncharacterized protein LOC103462027 isoform X2 | *Poecilia reticulata* |
| gi\|1039391228 | PREDICTED: uncharacterized protein LOC103462027 isoform X3 | *Poecilia reticulata* |
| gi\|1042344200 | PREDICTED: uncharacterized protein LOC108259564 | *Ictalurus punctatus* |
| gi\|1049223988 | PREDICTED: uncharacterized protein LOC108425742 | *Pygocentrus nattereri* |
| gi\|1049223998 | PREDICTED: uncharacterized protein LOC108425745 | *Pygocentrus nattereri* |
| gi\|1079739724 | PREDICTED: uncharacterized protein LOC108887947 | *Lates calcarifer* |
| gi\|1083437271 | PREDICTED: uncharacterized protein LOC108920476 | *Scleropages formosus* |
| gi\|1101587484 | PREDICTED: uncharacterized protein LOC109101583 | *Cyprinus carpio* |
| gi\|1109005738 | PREDICTED: uncharacterized protein LOC104919762 | *Larimichthys crocea* |
| gi\|1129966429 | PREDICTED: uncharacterized protein LOC109526012 isoform X1 | *Hippocampus comes* |
| gi\|1129966431 | PREDICTED: uncharacterized protein LOC109526012 isoform X2 | *Hippocampus comes* |
| gi\|1143364395 | PREDICTED: uncharacterized protein LOC109624947 | *Paralichthys olivaceus* |
| gi\|1168901793 | uncharacterized protein LOC109987033 | *Labrus bergylta* |
| gi\|1168961425 | uncharacterized protein LOC109965717 | *Monopterus albus* |
| gi\|1188118180 | uncharacterized protein LOC110161215 | *Boleophthalmus pectinirostris* |
| gi\|1211305958 | uncharacterized protein LOC110500300 | *Oncorhynchus mykiss* |
| gi\|1228998298 | uncharacterized protein LOC110960892 | *Acanthochromis polyacanthus* |
| gi\|1248995010 | uncharacterized protein LOC103024716 isoform X1 | *Astyanax mexicanus* |
| gi\|1248995021 | uncharacterized protein LOC103025024 | *Astyanax mexicanus* |
| gi\|1250101633 | uncharacterized protein LOC111224736 | *Seriola dumerili* |
| gi\|1308390568 | uncharacterized protein LOC111567341 | *Amphiprion ocellaris* |
| gi\|1316094177 | uncharacterized protein LOC102218441 isoform X1 | *Xiphophorus maculatus* |
| gi\|1316094179 | uncharacterized protein LOC102218441 isoform X2 | *Xiphophorus maculatus* |
| gi\|1317092885 | uncharacterized protein LOC111651667 | *Seriola lalandi dorsalis* |
| gi\|1338766414 | uncharacterized protein LOC111850233 isoform X1 | *Paramormyrops kingsleyae* |
| gi\|1338766416 | uncharacterized protein LOC111850233 isoform X2 | *Paramormyrops kingsleyae* |
| gi\|1338766418 | uncharacterized protein LOC111850233 isoform X3 | *Paramormyrops kingsleyae* |
| gi\|1338827765 | uncharacterized protein LOC111838144 isoform X1 | *Paramormyrops kingsleyae* |
| gi\|1338827767 | uncharacterized protein LOC111838144 isoform X2 | *Paramormyrops kingsleyae* |
| gi\|1357729487 | uncharacterized protein LOC112160901 isoform X1 | *Oryzias melastigma* |
| gi\|1357729489 | uncharacterized protein LOC112160901 isoform X2 | *Oryzias melastigma* |
| gi\|1367392994 | uncharacterized protein LOC112243723 | *Oncorhynchus tshawytscha* |
| gi\|1386862434 | uncharacterized protein LOC108250161 | *Kryptolebias marmoratus* |
| gi\|1389922954 | uncharacterized protein LOC103378686 | *Cynoglossus semilaevis* |
| gi\|1395229706 | Interleukin 1 beta-like 2 | *Scophthalmus maximus* |
| gi\|1395229707 | Interleukin 1 beta-like 2 isoform 2 | *Scophthalmus maximus* |
| gi\|1395229708 | Interleukin 1 beta-like 2 isoform 3 | *Scophthalmus maximus* |
| gi\|1468850528 | uncharacterized protein LOC113019000 | *Astatotilapia calliptera* |
| gi\|1469049901 | uncharacterized protein LOC113111075 | *Carassius auratus* |
| gi\|1469138939 | uncharacterized protein LOC113055294 | *Carassius auratus* |
| gi\|1469977669 | uncharacterized protein LOC113138041 isoform X1 | *Mastacembelus armatus* |
| gi\|1469977671 | uncharacterized protein LOC113138041 isoform X2 | *Mastacembelus armatus* |
| gi\|1473012870 | uncharacterized protein LOC113147655 | *Anabas testudineus* |
| gi\|1503285850 | uncharacterized protein LOC113547441 | *Pangasianodon hypophthalmus* |
| gi\|1503285852 | uncharacterized protein LOC113547442 | *Pangasianodon hypophthalmus* |
| gi\|307746681 | interleukin 1 beta | *Danio rerio* |
| gi\|307746683 | interleukin 1 beta | *Danio rerio* |
| gi\|339267712 | IL-1Ra protein | *Tetraodon nigroviridis* |
| gi\|339267714 | IL-1Ra protein | *Danio rerio* |
| gi\|348516653 | uncharacterized protein LOC100699119 | *Oreochromis niloticus* |
| gi\|410912848 | PREDICTED: uncharacterized protein LOC101066360 isoform X1 | *Takifugu rubripes* |
| gi\|422001752 | interleukin 1 beta-like 2 | *Paralichthys olivaceus* |
| gi\|422001754 | interleukin 1 beta-like 2 | *Paralichthys olivaceus* |
| gi\|47230630 | unnamed protein product | *Tetraodon nigroviridis* |
| gi\|498949373 | uncharacterized protein LOC101480448 | *Maylandia zebra* |
| gi\|548343437 | PREDICTED: uncharacterized protein LOC102205482 | *Pundamilia nyererei* |
| gi\|554866677 | PREDICTED: uncharacterized protein LOC102308236 isoform X1 | *Haplochromis burtoni* |
| gi\|554866679 | PREDICTED: uncharacterized protein LOC102308236 isoform X2 | *Haplochromis burtoni* |
| gi\|556983726 | PREDICTED: uncharacterized protein LOC102363048 | *Latimeria chalumnae* |
| gi\|571255618 | interleukin-1 family member | *Tetraodon nigroviridis* |
| gi\|583970305 | PREDICTED: uncharacterized protein LOC102783726 | *Neolamprologus brichardi* |
| gi\|594190782 | interleukin-1 family member A | *Danio rerio* |
| gi\|597793178 | uncharacterized protein LOC103024716 isoform X2 | *Astyanax mexicanus* |
| gi\|617426140 | PREDICTED: uncharacterized protein LOC103143562 isoform X1 | *Poecilia formosa* |
| gi\|617426143 | PREDICTED: uncharacterized protein LOC103143562 isoform X2 | *Poecilia formosa* |
| gi\|632948885 | PREDICTED: uncharacterized protein LOC103177481 isoform X1 | *Callorhinchus milii* |
| gi\|657574180 | PREDICTED: uncharacterized protein LOC103365194 | *Stegastes partitus* |
| gi\|736288474 | PREDICTED: uncharacterized protein LOC104963546 | *Notothenia coriiceps* |
| gi\|742167560 | PREDICTED: uncharacterized protein LOC105019922 | *Esox lucius* |
| gi\|742167564 | PREDICTED: uncharacterized protein LOC105019922 | *Esox lucius* |
| gi\|768935956 | PREDICTED: uncharacterized protein LOC101066360 isoform X2 | *Takifugu rubripes* |
| gi\|808861535 | hypothetical protein EH28 11529 | *Larimichthys crocea* |
| gi\|831287494 | PREDICTED: uncharacterized protein LOC105893267 | *Clupea harengus* |
| gi\|831287596 | PREDICTED: uncharacterized protein LOC105893317 | *Clupea harengus* |
| gi\|831555769 | uncharacterized protein LOC105934419 | *Fundulus heteroclitus* |
| gi\|928077817 | PREDICTED: uncharacterized protein LOC106536268 | *Austrofundulus limnaeus* |
| gi\|938072961 | hypothetical protein Z043 110336 | *Scleropages formosus* |
| gi\|961871480 | PREDICTED: uncharacterized protein LOC106952009 isoform X1 | *Poecilia latipinna* |
| gi\|961871484 | PREDICTED: uncharacterized protein LOC106952009 isoform X2 | *Poecilia latipinna* |
| gi\|973191081 | PREDICTED: uncharacterized protein LOC102690436 isoform X1 | *Lepisosteus oculatus* |
| gi\|973191083 | PREDICTED: uncharacterized protein LOC102690436 isoform X2 | *Lepisosteus oculatus* |
| gi\|974059250 | PREDICTED: uncharacterized protein LOC107082510 | *Cyprinodon variegatus* |
| TBA | Novel intereleukin 1 family member | *Cyclopterus lumpus* |
| **Other** |  |  |
| gi\|736184516 | PREDICTED: uncharacterized protein LOC104945633 isoform X1 | *Notothenia coriiceps* |
| gi\|736184519 | PREDICTED: uncharacterized protein LOC104945633 isoform X2 | *Notothenia coriiceps* |
