# Supplementary material for: Interleukin-1 Ligands and Receptors in Lumpfish (Cyclopterus lumpus L.): Molecular Characterization, Phylogeny, Gene Expression, and Transcriptome Analyses
Source: Front Immunol. 2020 Apr 2;11:502. doi: 10.3389/fimmu.2020.00502 (PMC7144542; doi:10.3389/fimmu.2020.00502)
Supplement: Supplemental Figure 5 — Phylogenetic tree of IL-1 ligands with accession numbers. The colors of the clades are similar to Figure 4. Bootstrap values > 80 % (of 100,000 iterations) are shown in the tree. The lumpfish sequences are written with red letters. [file Image_5.pdf]

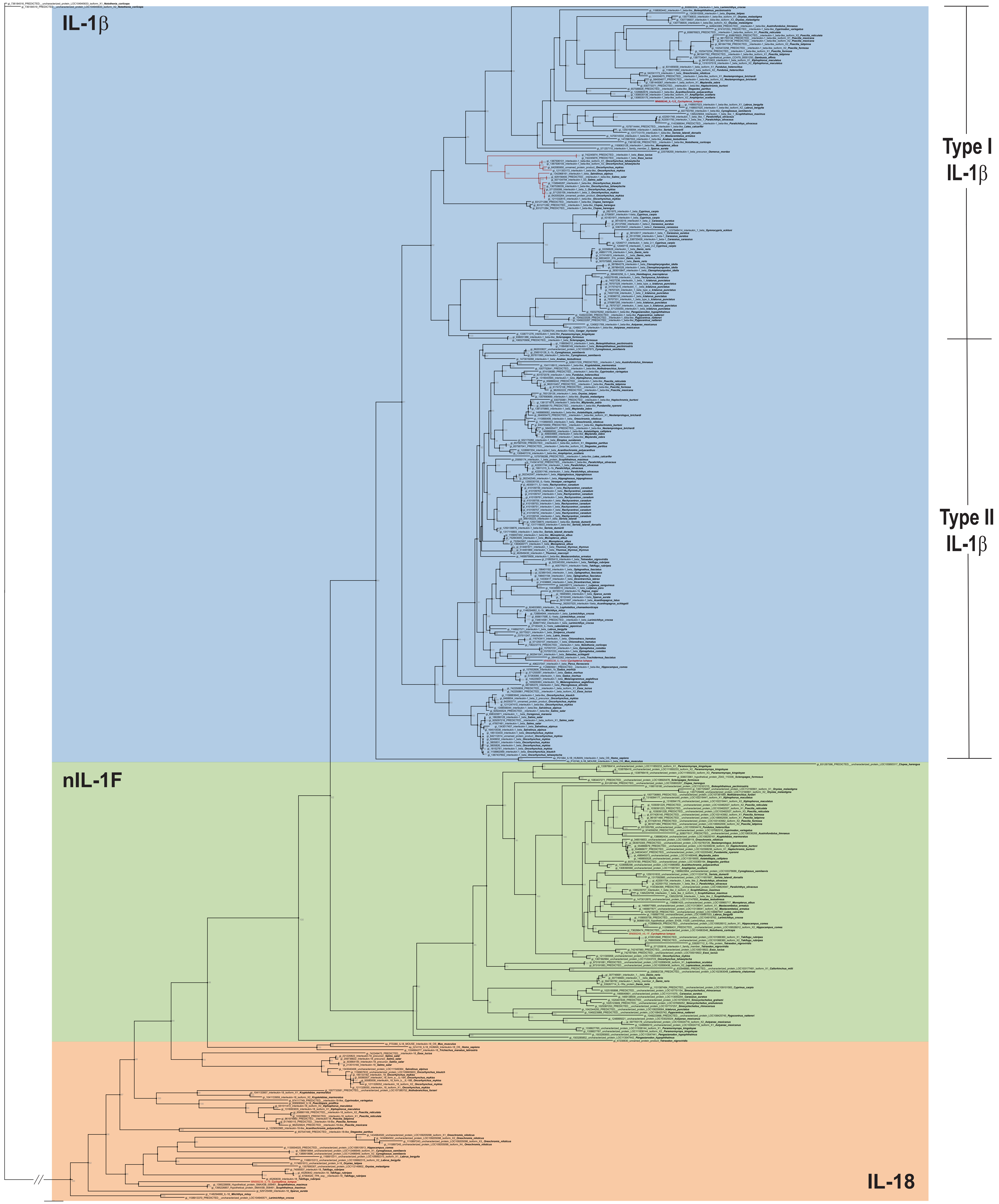

Suppl. Fig. 5
